# Optimal material selection of biocomposites for structural applications: an integrated fuzzy CRITIC-COPRAS approach

**DOI:** 10.1038/s41598-025-25622-z

**Published:** 2025-11-24

**Authors:** Ashish Soni, Sonu Kumar Gupta, Sethupathi Bose, Jitendra Kumar Katiyar

**Affiliations:** 1 Centre for Additive Manufacturing, Chennai Institute of Technology, Chennai, Tamil Nadu 600069 India; 2 Department of Civil Engineering, Institute of Engineering and Technology, Sandip University, Nashik, MH 422212 India; 3 Department of Mechanical and Industrial Engineering, Manipal Institute of Technology, Manipal Academy of Higher Education, Manipal, Karnataka 576104 India

**Keywords:** Biocomposites, Hybrid biofillers, Material selection, Mathematical model, Characterizations, Engineering, Environmental sciences, Materials science

## Abstract

The material selection is a decisive factor in the development and workability of composites. The research proposed an integrated fuzzy CRiteria Importance Through Intercriteria Correlation (CRITIC) and Complex Proportional Assessment (COPRAS) for material selection of 3D printed biocomposites developed by using fruit waste-derived biofillers and biodegradable polymers. The objective was to develop a mathematical model for materials selection of biocomposites for structural applications such as floor tiles, pavements, building blocks, doors, etc. Also, the composites can be effectively implemented in places where the consideration for mechanical and tribological performance under ambient conditions such as sliding elements, automobiles bumpers and interiors, frames, etc. are of prime importance The novelty of the work can be highlighted from the development of novel biocomposites and mathematical model which can effectively deals with the material selection problems in conditions of vagueness and hesitancy in the decision makers. The work will save time and resources and reduce the cost of biocomposites. The work brings sustainability to the composites manufacturing industries. CThe RITIC approach has revealed that the impact strength is the most significant criterion, whereas sliding wear is the least significant factor for the composites. The approach has identified the reinforcement of 20 wt.% of banana peel powder with 80 wt.% of polylactic acid, i.e. alternative A4, as the most suitable material for structural applications, whereas alternative A6 is composed of 90 wt.% of PLA and 10 wt.% of hybrid biofillers of banana peel powder and orange peel powder (in equal proportions), is the least preferable alternative. The identified best composite has demonstrated an impact strength of 20,000 kJ/m^2^ and a specific wear rate of 0.00066 mm^3^/N-m. Moreover, the composites have obtained an optimal hardness of 82.2 (shore D) and a good compressive strength of 68.81 MPa. The proposed integrated CRITIC-COPRAS approach has identified the ranking sequences of the alternatives as A4 > A7 > A8 > A1 > A2 > A5 > A3 > A6. The work has successfully developed a mathematical model to assist the materials selection process of eco-friendly composites and reduce the associated problems due to the mismanagement of plastics and agro-industrial wastes.

## Introduction

The rising cost of conventional materials, dwindling natural resources, concern for environmental pollution, and technological advancement have prompted the world towards novel and eco-friendly materials. Plastics are widely used materials in different sectors, from households to industries. Due to the increasing usage of plastics in different sectors such as food packaging, construction, transportation and electrical/electronic markets. The global production of plastics is estimated to reach about 368 million tons (Mt) per year ^1^. It is anticipated that about 53 Mt of plastic waste will be present in the ecosystem by the year 2030, and the amount will quadruple by the year 2050 ^2^. Despite several remedial efforts to restrict the usage of plastics, it can’t be completely arrested due to their diverse applications and beneficial properties. The existing inadequate practices of managing plastic waste and ineffective recycling of plastic waste make the management of plastic waste a global challenge to the environment and public health. It is estimated that about 22% of the globally generated plastic is mismanaged, while about 19% of the plastics are being incinerated, 49% is disposed of as landfill, and only a small fraction of about 9% is recycled ^3^. The improper management of plastic waste has created environmental challenges, which can be reduced by the use of biodegradable polymers.

Biopolymers degrade into microplastics in the natural environment, which further interact with the organic matter present in the soil ^4^. The researchers have utilized different plant-based reinforcements in biopolymers for the development of eco-friendly materials. The evaluations of the different properties have found that jute/hemp reinforced PLA composites have exhibited better properties as compared to other composites. The jute/hemp reinforced PLA composites are found to decompose at 250 °C and start losing weight at 350 °C ^5^. The comparisons of the erosive wear for the composite developed by reinforcement of Ipomoea staphylina with epoxy, vinyl ester and PLA matrix have found a lower erosive wear for the epoxy based composites as compared to the vinyl ester and PLA based biocomposites ^6^. The mechanical properties of the PLA based biocomposites were found to be influenced by the stacking sequences of the fibers ^7^. The jute based natural fiber composite has demonstrated its potential for industrial applications ^8^. The baggase fiber has shown its potential for the development of biocomposites for different applications. The bagasse fiber possesses a good tensile strength, Young modulus and requires a temperature of 200–240 °C for decomposition. The compatibility of fiber with polymeric matrix is influenced by cellulose and the dielectric constant. The treatment of fibers can influence the workability of the composites ^9^. Artocarpus hirsutus (AH) fiber has been utilized as an effective filler for the development of bamboo fiber (NF)/polyethylene (PE) biocomposites. The investigations of the properties have found a better flexural and tensile strength for the composite with 20 wt.% of the NF and 3 wt.% of cellulose ^10^. The work has investigated the development of palm oil based fiber for the development of polyester hybrid composite. The mechanical properties of the composite were investigated as per the ASTM standards. The results have highlighted the potential of the developed composite in biomedical applications ^11^. The carbon fiber was utilized in epoxy resin for the development of composites for biomedical applications. The interfacial bonding between the fiber and matrix was improved by increasing the surface roughness of the carbon fibers by oxidizing the fibers with acid. It was found that the oxidation of the fiber increases the strength, also the longer fiber has demonstrated a higher tensile strength ^12^. The researcher has developed composites by using shell powder of wood apple and coconut in different proportions. The mechanical and tribological properties of the composites improve with the reinforcement^13^. The work has explored the potential of agricultural wastes in the development of sustainable composites. It was suggested that the fine-tuned natural resin and dual-fiber reinforcement can significantly improve the performance of the composites^14^. The work has summarized the advancement in the prosthesis design with special attention on the dual mobility bearings to improve the stability and range of motion, and polymer-based bearing for the enhancement in the durability and reduction in the sliding wear ^15^. The work has studied the deformation and wear responses of the three different types of materials namely, metal ceramic and diamond by using Finite Element Analysis (FEA). A two dimensional model was simulated, then compared running and volumetric wear, which shows less deformation and wear for hard-on-hard bearings ^16^. The work has evaluated the Tresca stress in CoCrMo-onCoCrMo hip implant on the basis of the mass index from patient and load variations by using FEA, which has found an increase in the Tresca stress and its distribution with the mass index ^17^. The researchers have used Abaca fiber in biopolymers for the development of eco-friendly material. The surface modification of the fibers by potassium permanganate has improved the crystallinity, while the hydrophilicity is reduced by the treatment with sodium hydroxide solutions ^18^.

Polylactic acid (PLA) is a biodegradable thermoplastic polyester made from renewable sources such as corn starch, sugarcane and cassava. Besides biodegradability, PLA has been known for its excellent physical and mechanical properties and exhibits excellent stiffen, making it a satisfactory candidate for biocomposites ^19^. It has attracted considerable interest due to its eco-friendliness, ease of processing and cost advantages over petroleum-based plastics. Nevertheless, its natural brittleness, lower thermal resistance and moisture sensitivity restrict its performance in challenging conditions. In order to address these limitations, PLA is frequently reinforced with natural fibers, nanoparticles, or blended with other polymers, improving its mechanical, thermal, and barrier characteristics. PLA has been successfully employed in the development of 3D printed biocomposites for the development of materials for wide applications, and the performance of the developed materials depends on the printing parameters and compositions. This research has enhanced the performance of biocomposite filaments made of PLA, magnesium (Mg), and polyethylene glycol (PEG) intended for bone scaffold applications through 3D printing. By employing Response Surface Methodology, the extrusion parameters such as temperature, screw speed, and the ratios of Mg and PEG were fine-tuned to produce a uniform filament diameter of 1.75 mm, complying with 3D printing specifications^20^. The work has identified the potential of graphene and SiO_2_ nanofillers to enhance the mechanical and fracture performance of epoxy and PLA composite for biomedical applications, which reveals that the addition of 0.15 wt.% of nanofillers enhances the mechanical properties of composites as compared to the neat epoxy-PLA ^21^. The researchers have successfully developed a biocomposite by utilizing PLA with the reinforcements of treated fiber and biochar from the Washingtonia filifera (WF) plant, and the evaluations of the properties were performed by dynamic mechanical and thermo-mechanical analyses ^22^. Moreover, the biochar and biomass derived from palm fibers were reinforced in PLA for the development of a biocomposite. The treatment of the fibers has successfully improved the performance ^23^. The investigations on the workability of the composite developed by using PLA and Washingtonia robusta palm tree and biochar powder having a size of 0.6 µm obtained due to the carbonization at 300 °C^24^. The investigations for the effect of chemical treatment, i.e. 10 wt.% of NaHCO_3_ for the different durations on the physical and mechanical properties has found an improvement in the tensile, impact and flexural properties with the treatment of fibers ^25^.

The study has explored the properties and applications of kenaf, sisal, pineapple, bamboo and banana fibres as natural reinforcement for the development of composites. The investigations on the mechanical properties have highlighted the potential application in industries which creates eco-friendly alternatives over synthetic fibres ^23^. The hybrid- fibers of jute fibres and waste duck feather were utilized as a reinforcement with polymer matrix in composites for structural and non-structural applications. The developed biodegradable products are helpful in reducing carbon footprint. The composites were developed by using three layers of duck feather and jute fibre which exhibited better mechanical performance compared to two-layer duck feather composites ^26^. The optimization of biocomposites can be done by varying resin structures and fiber frameworks, along with utilizing jute, cotton, coir and silk. This is a convenient way to produce biocomposites due to their non-polluting, renewable and sustainable characteristic ^27^. This study has proposed the examination of natural fibres such as lemongrass and vetiver as fillers in composites that act as a sustainable alternative for various applications. These composites are eco-friendly and possesses a better mechanical properties, thermal behaviour, and surface features ^28^. The study has examines the effect of stacking sequence in the mechanical, tribological and morphological characteristics of grave-jute composites ^29^. A scientific investigation is carried out to study the mechanical properties, morphological properties of vetiver-jute fibre hybrid composites. The composites are created by arranging the fibers in different layers with epoxy resin. Notably, Jute-Vetiver-Jute hybrid composites has obtained a better mechanical and morphological properties than the neat and hybrid epoxy composites suggesting significant potential for various industrial applications ^30^. This study has investigated the effect of volume fraction of natural fibers in composites. The findings highlight the viability of using these fibers as sustainable reinforcements in 3D printed composites for sectors like automotive, aerospace and biomedical applications ^31^. This study has explored the use of natural fibre composites having applications in the automotive sector ^32^. This study has summarizes the work on mechanical, chemical, thermal, acoustical and morphological properties of fibre reinforced composites which reveals the potential applications of the chicken feather fibre (CFF) in different commercial industries for the advancement of eco-friendly sustainable products ^33^. A compressive review was performed on the properties and application of natural fibres therefore contributing in sustainable development. The study focuses on manufacture on green composites and the growing applications of these fibre in 3D and 4D composites. The study promotes the use of natural fibres over synthetic fibres and supports the eco-friendly products across a variety of industries ^34^. The work investigates the effect of fibre orientation on the mechanical and morphological qualities of jute-acacia hybrid composites. The sandwich composites outperformed two-layer stack patterned composites in mechanical and morphological qualities, implying potential applications across industries ^35^. The study has used ultrasonication and compression molding to produce Multi-walled Carbon nanotubes (MWCNT)-epoxy composites under different five (05) formulations of 0 (neat), 0.5, 1, 1.5, and 2 (wt. %) with a thickness of 5.0 ± 0.1 mm. The was revealed that composites having 0.5 wt.% of MWCNT has obtained the highest performance while the higher concentrations negatively impacting characteristics. This research is important for applications which require outstanding mechanical, tribological and morphological properties in the structural, aerospace, and automotive industries^36^.

3D printing or additive manufacturing fabricate the components by layer by layer technique. The technique can fabricate intricate and customized lightweight structure which are not possible by any conventional methods. The technique eliminates the wastage of materials and offer an optimal utilization of the resources. The technique save time and enables the development of functionally graded composites which are difficult by any other technique. The 3D printing offers new applications in biomedical, aerospace, construction, and consumer goods 3D printing was employed for the fabrication of optical oxygen sensor through the integrations of silicon matrix and oxygen sensitive dyes. The high performance and biocompatibility of silicon matrix and effectiveness of sensor components has significantly favoured their development as future healthcare material ^37^. The work has demonstrated the implementation of FEA for the improvement in the precisions and accuracy of surgery. FEA has assisted in the customization of the implants and evaluations of the surgical techniques. The modern imaging techniques like augmented and virtual reality has improved the precision of hip arthoplasty ^38^. The researcher has developed different clean aligners of different thickness by using 3D printing advanced scanning technologies. The research findings has provided a valuable insight for the optimizations of mechanical performances ^39^. The manufacturing of interference screws by using 3D printed biocomposite filaments was performed by using varying the nozzle temperature and printingspeed. It was observed that the bonding of the layer is degraded by a higher nozzle temperature and printing speed with the increase in the porosity and decrease of the mechanical strength ^40^. The different loading fractions of Acacia nilotica fibers of 0, 2, 4 and 6 (wt.%) has been successfully reinforced with epoxy in the development of composite. The loading fractions of 6 wt.% of Acacia nilotica fibers at 20 N has decreases the wear rate and coefficient of friction of the composite by 61.2% and 34.3%, respectively ^41^. The recycling of polyurethane has been performed by incorporation with PLA. The composites were developed by using 3D printing technique and the investigation of the mechanical properties has found that the reinforcement of 3 wt. % of polyurethane has successfully increases the mechanical properties of the composites ^42^. The researcher have developed a novel 3D printed composite by using the stacking sequences of the two different types of natural fibers with PLA and it was revealed that the mechanical properties of the composites is influenced by the stacking sequences of the fibers and resulted the optimal tensile strength of 74.82 MPa ^43^. The particles of M. citrifolia bark has been successfully reinforced with 0, 3 and 6 (wt.%) of PLA in the development of 3D printed composite. It was found that the loading of 6 wt.% has significantly improved that tensile strength ^44^. The incorporation of 2 wt.% of Tamarindus indica in PLA has improved the tensile strength, compressive strength and flexural strength of the composites by 12.64% and 19.87% and 15.57%, respectively ^45^. Moreover, the incorporations of two biofillers namely, lignin and spent coffee grounds in PLA has successfully improved the performance and printability of the 3D printed composites^46^.

The awareness of the public about healthy foods has significantly increased the demand for fruits. The Food and Agriculture organization of the United Nations has estimated the global production of fruits to be about 1.6 billion tons which generates a massive quantity of fruit waste ^47^. The different fruit processing sectors, such as hotels, restaurants, juice manufacturers, etc., generate a considerable amount of fruit waste. The disposal of fruit wastes in composting facilities, open dumping sites and recycling facilities contaminates the land and water resources. The transformation of fruit wastes into a valuable resource is a multifaceted approach for social, economic and environmental benefits and favors the promotion of recycling of fruit wastes into value-added products ^48^. The fruit waste constitutes a significant portion of the agricultural waste, such as banana peel, avocado peel, citrus processing wastes, etc. Due to the higher health benefits and nutritional value, the consumption of citrus fruits is more than that of other fruits ^49^. The countries like India, China, the USA, Brazil, Spain and Mexico are the leading producers of citrus fruits ^50^. The extraction of orange juice generates orange processing wastes, where orange peel constitutes approximately half of the residue. The peel of orange consists of various advantageous components such as cellulose, pectin, essential oil and soluble sugar which are valorized for versatile applications ^51^. The orange waste is generally managed by incineration, composting and landfills which have several advantages and limitations. Due to the high moisture content in orange peel, incineration is not considered a beneficial approach from the eco-environmental point of view. The development of value-added products from orange peels opens avenues for fruit industries and promotes sustainability. The orange peel powder is gaining importance as a biofiller in the development of biocomposites. The wide availability, low-cost, biodegradability and its potential to improve the properties in biocomposites are the notable attributes of biofillers ^52^. Banana is the second most widely cultivated fruit in the world and occupies about 16% of the global production of fruits ^53^. The global production of the banana is reported to about 115.74 million metric tons and countries like India, Brazil, China, Colombia, etc., are the leading producers of banana ^54^. The banana peel constitutes about 30–40% of the total banana fruit which has generated about 34.72–46.29 million metric tons of banana peel in 2018 ^55^. The powdered form of banana and orange peel can be an effective biofillers for PLA due to their status as lignocellulosic agricultural waste offering complementary chemical properties (cellulose, hemicellulose, lignin, and notable pectin content) that can enhance stiffness and biodegradability while reducing material costs and carbon emissions^56^. Rich in cellulose/hemicellulose and pectin, banana peel provides solid particulate reinforcement and has been demonstrated to enhance specific modulus and tribo-mechanical performance when blended with PLA-based systems. Orange peel is composed of pectin, cellulose, polyphenols and volatile limonene which serve as particulate fillers and can improve the biodegradation and antioxidant properties of PLA composites ^57^. However, due to PLA’s relatively hydrophobic nature and the hydrophilic characteristics of these peels, weak interfacial adhesion may occur; thus surface treatments or compatibilizers, such as maleic-anhydride grafted PLA or silane treatments are often used to enhance dispersion, stress transfer, and the resulting tensile and flexural properties ^58^. Collectively, these chemical and interfacial aspects position banana and orange peel powders as appealing, cost-effective biofillers for PLA with established mechanical and tribological advantages highlighted in recent research.

The concern for environmental challenges has stimulated the world to promote biocomposites^59^. The industrialists and researchers have strived towards eco-friendly materials for the promotion of recycling facilities to reduce the burden of waste and the demand for resources ^60^. Biocomposites are made from recyclable materials which can be effectively disposed of without causing any harm to the environment. Recently, a considerable improvement has been made in the development of biocomposites for diverse applications ^61^. The biocomposites offer several benefits over conventional composites such as the potential to reduce CO_2_, improved performance, carbon neutrality, etc. ^62^. The urge to sustain in the manufacturing sectors along with the growing concern for environmental health has compelled the world to pioneer novel and eco-friendly materials for the development of high-performance and sustainable composites. The conventional composites are being replaced by sustainable and environmentally friendly materials. The incorporation of biofillers overcomes the limitations of the polymeric matrix in biocomposites by increasing the interfacial adhesion between the fillers and matrix through chemical bonding and mechanical interlocking to assist in stress transfer ^63^.

The material selection is one of the crucial steps in the development of any product and is performed during the design phase ^64^. The proper selection of material increases the overall product life and ascertains the reliability of any product, whereas an improper selection of material can lead to the failure of the product and adversely affect the performance, efficiency and overall lifecycle ^65^. The dependency of product performance on several factors such as strength, safety, economic, environmental, etc., has increased the complexity in material selection. Moreover, the design requirements and objectives are conflicting and require the prioritization of the attributes. The selection of suitable materials for optimal performance requires a multi-dimensional trade-off among the different properties ^66^. The selection of materials by hit and hit-and-trial method is cumbersome and results in the wastage of resources and time. The wide availability of materials and their responses has recognized material selection as a complex decision-making approach ^67^. To overcome the challenges in material selection, a systematic and logical approach is required to identify the key criteria which influence the material selection for engineering applications.

The application of MCDM techniques for material selection has gained significance. The MCDM technique addresses the complexity of material selection by systematically weighting the criteria and eliminating the conflicting factors. Based on the complexity of the problem and criteria, there are different MCDM techniques available for material selection each having a unique benefits and limitations. The implementation of an analytic hierarchy approach for material selection of oil and gas pipelines has identified TA36 as the most suitable material for submarine pipelines ^68^. The researchers have implemented Pythagorean fuzzy soft sets (PFSSs) for handling the ambiguity and uncertainty in material selection problems ^69^. Xie et al. have proposed the theory of probabilistic interval-valued hesitant fuzzy set—A technique for order preference by similarity to an ideal solution (PIVHFS-TOPSIS) to deal with the hesitancy in the material selection of 3D printed composites. The identified optimal material has demonstrated its potential application in automobile chassis ^70^. The implementation of the entropy-based VIseKriterijumska Optimizacija I Kompromisno Resenje (VIKOR) method for the identification of a suitable dental restorative composite has demonstrated that the mathematical model which can effectively address the challenges in material selection for biomedical applications ^71^. A unique framework work was proposed through the integration of Interval-Valued Neutrosophic Sets (IVNSs) with the entropy–Multi Atributive Ideal-Real Comparative Analysis (MAIRCA) for the identification of a sustainable material for spar application and a good correlation of the proposed approach with other MCDM methods was demonstrated ^72^. The entropy weight and TOPSIS were successfully implemented for the selection of filler materials and identified recycled ceramic derived from hard coal ashes as a suitable filler for energy storage systems ^73^. Multi-Objective Optimization based on Ratio Analysis (MULTIMOORA) and Weighted Aggregated Sum Product Assessment (WASPAS) were successfully implemented for the selection of phase change material under seven (07) different criteria which found that the thermal conductivity is the most significant factor for a phase change material and identified graphite as a suitable phase change material ^74^. The implementation of VIKOR for the selection of a suitable organic material for solar drying has identified polyethyne glycol as the worst material by all the techniques considered in the study, whereas palmitic acid is considered as the worst material by the combined assessment technique ^75^. A novel three-way decision model under an interval-valued triangular fuzzy number (IVTFN) was implemented for the material selection of additively manufactured composites ^76^. A suitable multiwall carbon nanotube from recycled plastic waste was selected by the implementation of multi-criteria decision-making techniques ^77^. The implementation of a multi-criteria decision-making problem for material selection has identified silicon carbide as the most suitable material for a milling cutter ^78^. The researchers have implemented Criteria importance through inter-criteria correlation (CRITIC)—Multi-Attributive Ideal-Real Comparative Analysis (MAIRCA) approach for material selection of phase change material for solar distillation systems and identified paraffin wax as the most suitable phase change material ^79^. The researchers have implemented Criteria Importance Through Intercriteria Correlation (CRITIC)-Measurement of Alternatives and Ranking according to Compromise Solution (MARCOS) for ranking of natural fibers reinforced sustainable composites for tribological applications ^80^. An integrated Analytical Hierarchy Process (AHP)-Technique for Order of Preference by Similarity to Ideal Solution (TOPSIS) was implemented for materials selection of natural composite under four (04) different criteria and has identified PLA as a suitable candidate for the development of composites for a two-stroke marine engine ^81^.

The graph theory and matrix approach were implemented for material selection of high-temperature thermochemical storage, and found that the approach can effectively recover material from high temperatures ^67^. The work has demonstrated the implementation of the Define, Measure, Analyze, Improve and Control (DMAIC) approach, and Grey Regression Analysis (GRA) for the selection of the most suitable matrix in natural fiber composites for helmets ^82^. Multiple-TRIangles ScenarioS (MUTRISS) with two scenarios were effectively applied to deal with the issues of material selection and have verified that the first scenario of MUTRISS is more reliable than the second scenario ^83^. A novel multi-criteria decision-making approach was implemented by using the Ansys Granta Edupack for the material selection of toys ^84^. An integrated AHP**-** Function Analysis System Technique (FAST) was successfully applied for material selection of pipe based on the cost, quality and function ^85^. A new hybrid framework consisting of Modified Digital Logic (MDL) and Measurement of Alternatives and Ranking according to Compromise Solution (MARCOS) under an interval-valued intuitionistic fuzzy (IVIF) environment implemented for the selection of microwave absorbing materials, has found that carbon nanotubes/Fe as the most suitable material for absorbing microwaves ^86^. The work has presented a hybrid Pythagorean fuzzy multiple criteria group decision-making methodology for material selection of additively manufactured composites ^87^. Response surface method (RSM) was employed to investigate the influence of weight of abaca, red mud and size of red mud on the sliding wear of the composites. The developed fuzzy model can predict the accuracy of the sliding wear of the composite with an accuracy of 87% ^88^.

The extensive literature has demonstrated that several MCDM techniques are widely employed for material selection in different engineering fields but there is still a lack of work on biocomposites especially for comparative studies on biofillers reinforced materials for structural applications such as pavements, frames, panels, etc. The application of a fuzzy-based mathematical model for the identification of a suitable composition for biocomposites developed by using orange and banana peel powder, and polylactic acid through additive manufacturing has not yet been demonstrated. In order to address the above research gaps, the present work proposed an integrated TFWBMAO based fuzzy CRITIC-COPRAS mathematical model for the material selection of biocomposites. CRITIC evaluate the weight of the criteria by considering the degree of conflict eliminating subjective bias then the COPRAS ranks the alternative. The combination of the framework has effectively balances subjectivity with statistical objectivity resulting in consistent and accurate results ^89^. The integrated TFWBMAO-based Fuzzy CRITIC-COPRAS approach is a combination of multiple advanced decision making approaches and warrant robust, and reliable results therefore, provides an effective and scientifically correct methodology for materials selections^90^. The study has utilized triangular membership functions to quantify the fuzziness while TFWBMAO operator effective handles the hesitancy among multiple experts which ensures that subjective, uncertain, and hesitant judgments are robustly integrated into the CRITIC–COPRAS framework. The objective of the research was not only to deal with the problems of material selection but also to rigorously validate the reliability and accuracy of the proposed model. Apart from its direct contribution to the material selection of biocomposites, the research presented an insight for the development of eco-friendly materials for wide applications. This integrated fuzzy based technique ranks the alternative and identifies a suitable composition for the development of sustainable and eco-friendly composites. The other traditional methods for material selection fails to handle vagueness and complexity the Triangular Fuzzy number based CRITIC-COPRAS approach provide a systematic and robust approach for identifications of a suitable compositions. The work will improve the implementation of eco-friendly composites for diverse engineering fields besides improving the accuracy of the decisions making in material selection. While the approach is novel the result is highly depends on the quality of the information or input. Additionally, although the approach is mathematically rigorous but the complexity of the approach makes it difficult for the adoption in the real-world system and it might oversight some of the critical aspects long-term performance. The remaining part of the work is formulated as follows: Section 2 gives the details of the fabrication and characterization of the biocomposites. Section 3 discusses the mathematical techniques and prerequisites used in this work. Section 4 gives the details of the proposed mathematical model. The application of the proposed mathematical model is presented in Section 5, then results are discussed in Section 6, and finally, in Section 7, the conclusion is derived and the future scope of the work is presented.

## Materials and methods

In this section, discussions of the raw materials and techniques for the development of biocomposites are provided. Also, the details of the characterization techniques are provided.

### Materials

**Fig. 1:**
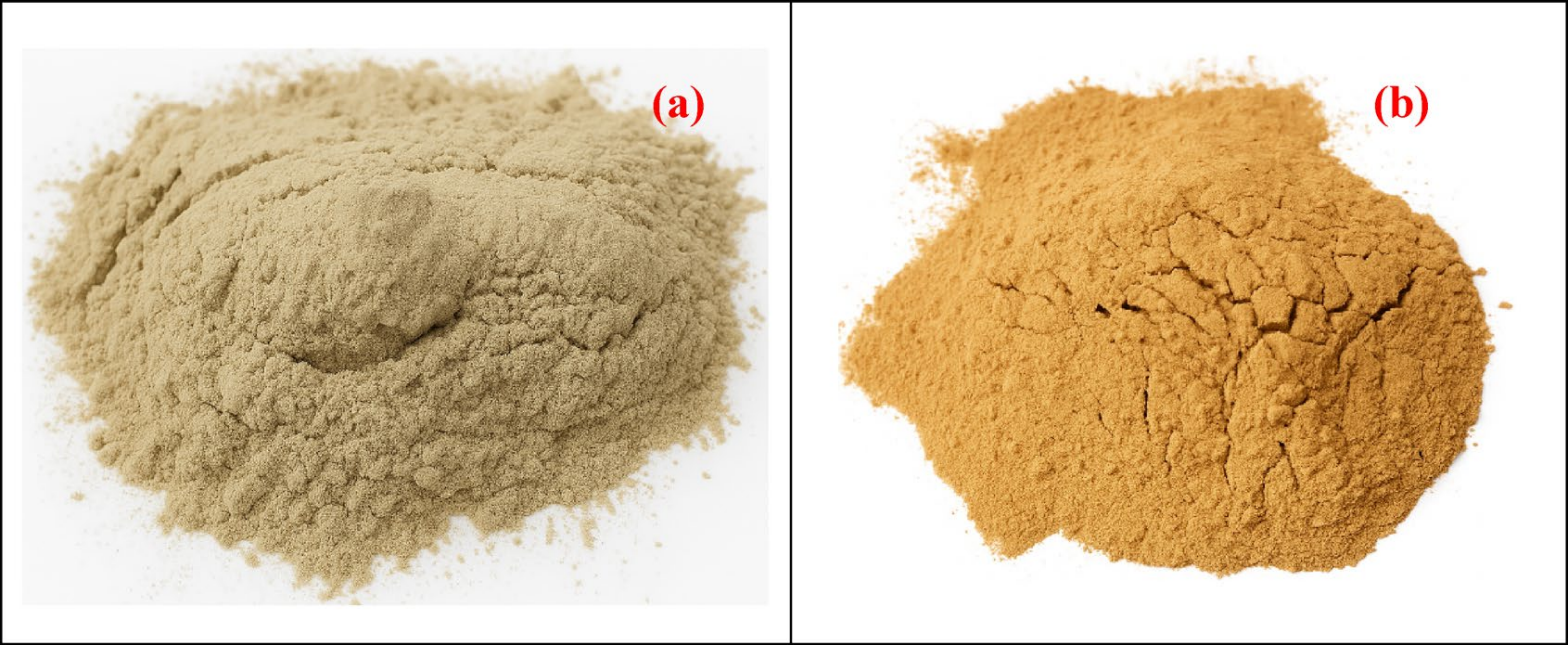
Images of biofillers (**a**) Orange peel powder, (**b**) Banana peel powder.

In the present study, PLA and biofillers are used as raw materials for the development of biocomposites. PLA pellets supplied by Natur Tec India Private Limited are used as matrix material; Orange and banana peel powder are used as biofillers in the development of biocomposites. PLA is a biodegradable semi-crystalline thermoplastic which is developed from the monomer lactic acid through poly-condensation. The density and melting point of the PLA are 1210–1430 kg/m^3^ and 150–160 (°C), respectively ^91^. The biofillers are prepared by collecting the peels of bananas and oranges from the local market. The collected peels of banana and orange were cleaned thoroughly to remove any moisture and alkali-treated to facilitate compatibility with the polymers. The obtained peels are dried completely to remove any moisture and crushed into powder form by using a ball miller to obtain the biofillers of Orange peel powder (OPP) and Banana peel powder (BPP) having a size of 100 $\mu m$ as shown in Fig. 1a, b, respectively^92^. The fillers size is measured by sieve analysis where a set of standard test sieves are arranged in decreasing order of the size. The fillers are mechanically vibrated for 5–10 min and the mass retained over the sieve is collected.

### Fabrication of biocomposites

**Fig. 2:**
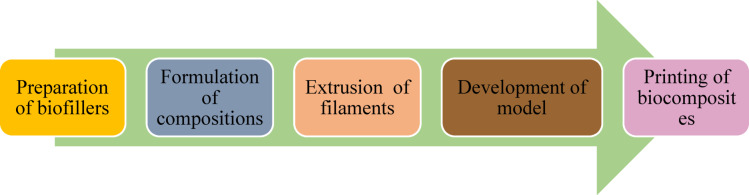
Process flow chart for 3D printing of the samples.

**Fig. 3:**
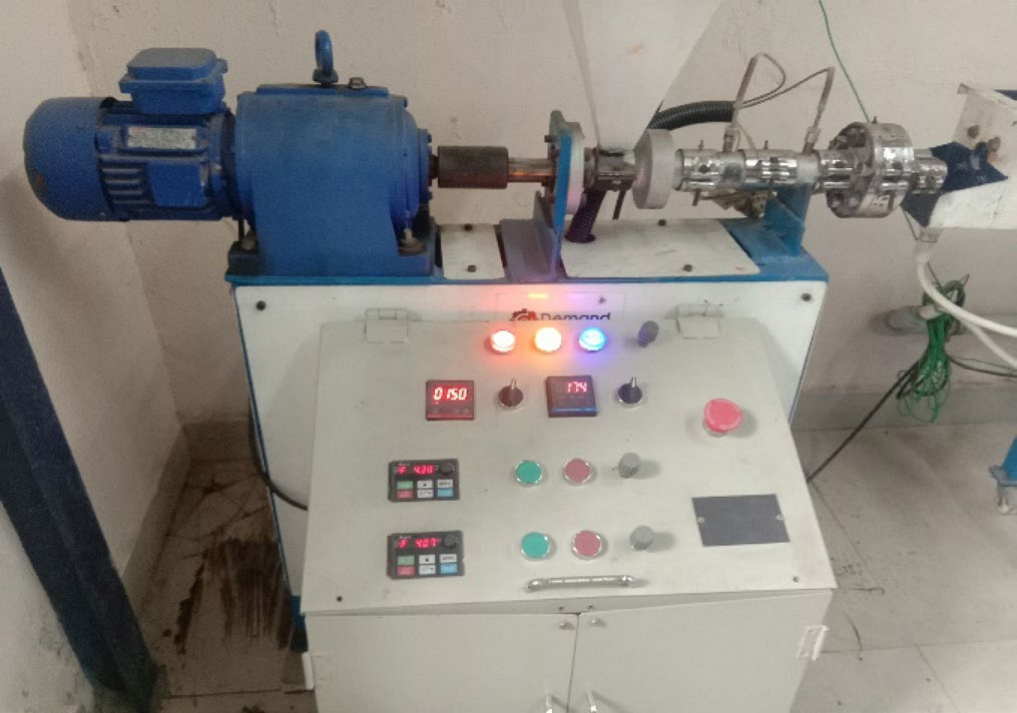
Extrusion of biofillers/PLA filament.

**Fig. 4:**
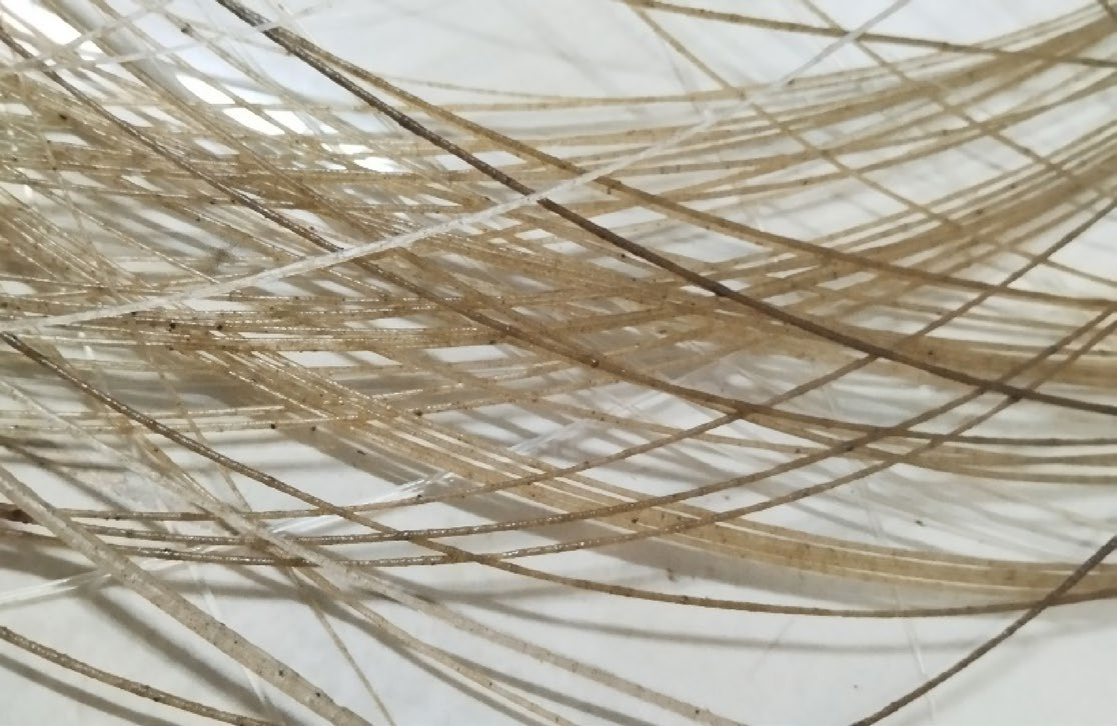
Image of the developed biofillers/PLA filament.

**Fig. 5:**
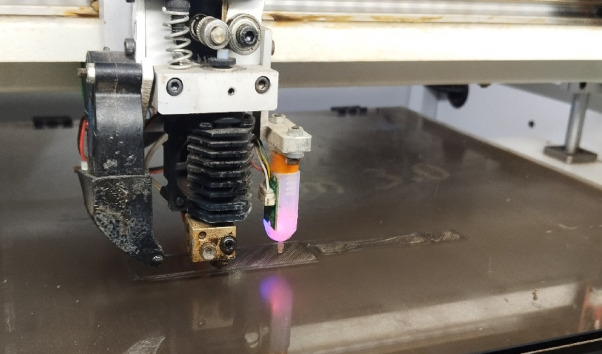
Images of the 3D printing of the samples.

The biocomposites are developed by using a 3D printing or additive manufacturing technique by following the compositions given in Table 1. The technique employed a layer-by-layer process to create a product from a digital file. The process flow chart for the fabrication of biocomposite is given in Fig. 2. The loading fractions of the fillers are taken by considering the fabricability of the filaments as beyond 20 wt.% of fillers there is brittleness in filaments making the printing difficult. Moreover, in order to explore the effect of hybrid biofillers the fractions BPP is equally replaced by OPP. The filaments are developed by using a filament extruder having a cylinder diameter of 45 mm and a nozzle diameter of 3 mm as shown in Fig. 3. The PLA are heated to a preheat temperature of 175 °C, and the extrusion temperature is maintained at 185–190 (°C). The filaments are allowed to cool by using a pool of water. The obtained filament of diameter 1.75 ± 0.05 mm as shown in Fig. 4 is used to print the object by using a 3D printing machine. The raster angle was set to + 45°/ − 45° and printing speed of 40 mm/s is taken for printing the biocomposites. The temperatures of the printing nozzle build platform were 220 °C and 60 °C, respectively. The diameter of the printing nozzle is 0.4 mm and thickness of the layer is 0.2 mm with infill density of 100%. The 3D model of the object is created by using a design software and converted into a Standard Tessellation Language (STL), then send to the 3D printer for the development of biocomposites as shown in Fig. 5.

**Table 1:** Details of the alternatives.

Alternative	BPP (Wt.%)	OPP (Wt.%)	PLA (Wt.%)	Sample designation
A1	5	0	95	B5P95
A2	10	0	90	B10P90
A3	15	0	85	B15P85
A4	20	0	80	B20P80
A5	2.5	2.5	95	P95BO5
A6	5	5	90	P90BO10
A7	7.5	7.5	85	P85BO15
A8	10	10	80	P80BO20

### Characterization

**Fig. 6:**
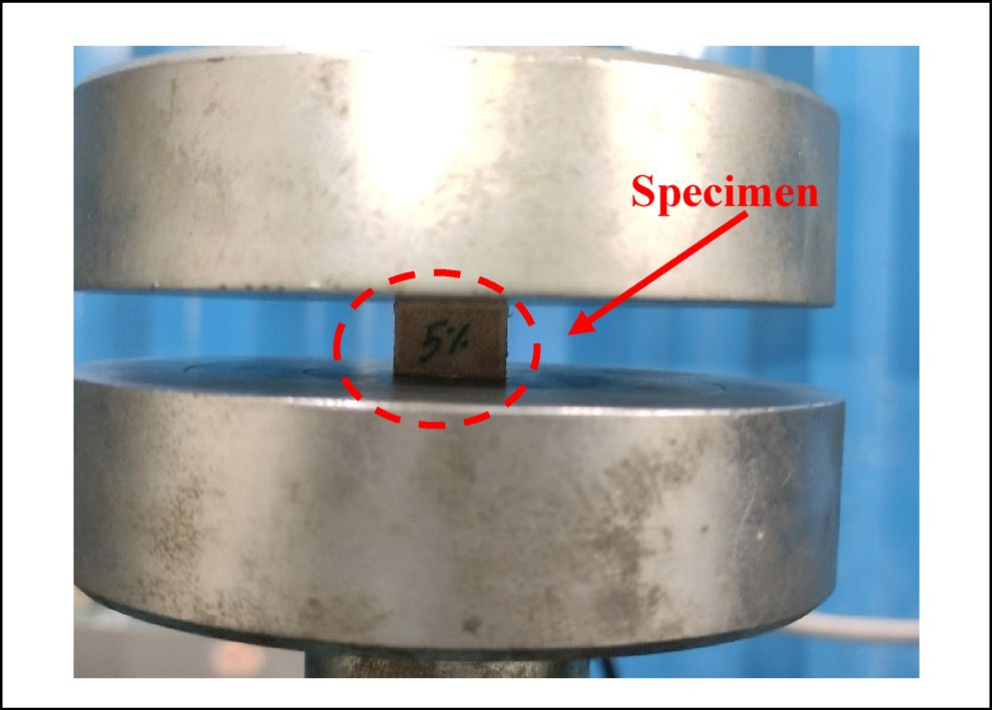
Images of the 3D specimen in compressions test.

**Fig. 7:**
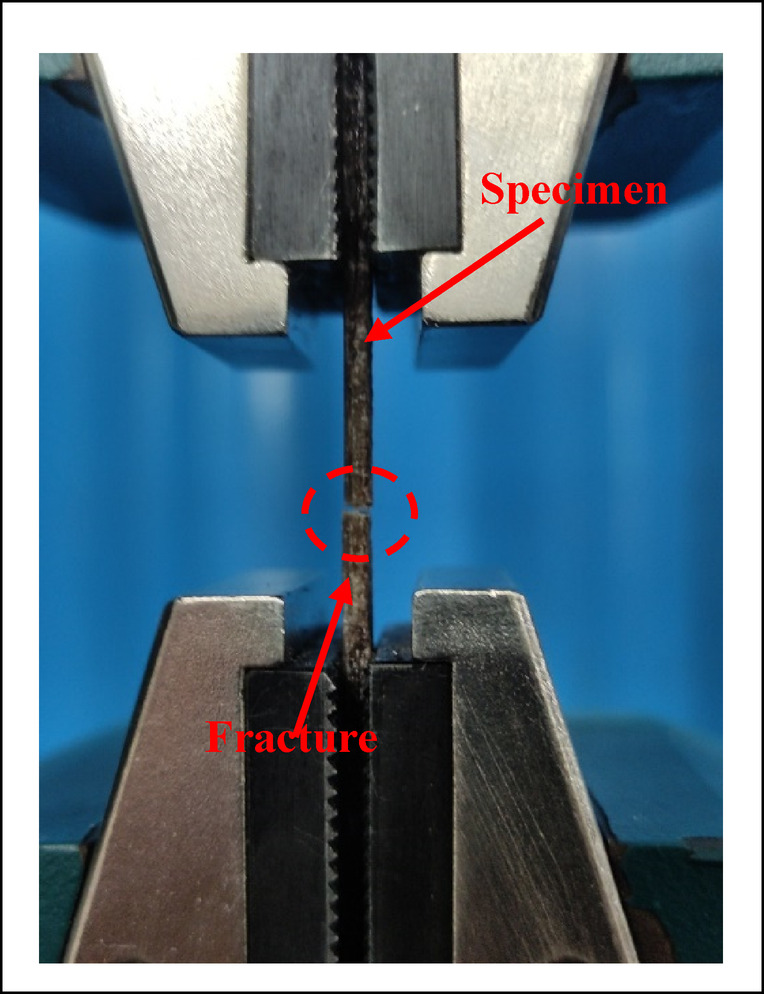
Images of the 3D specimen in tensile test.

**Fig. 8:**
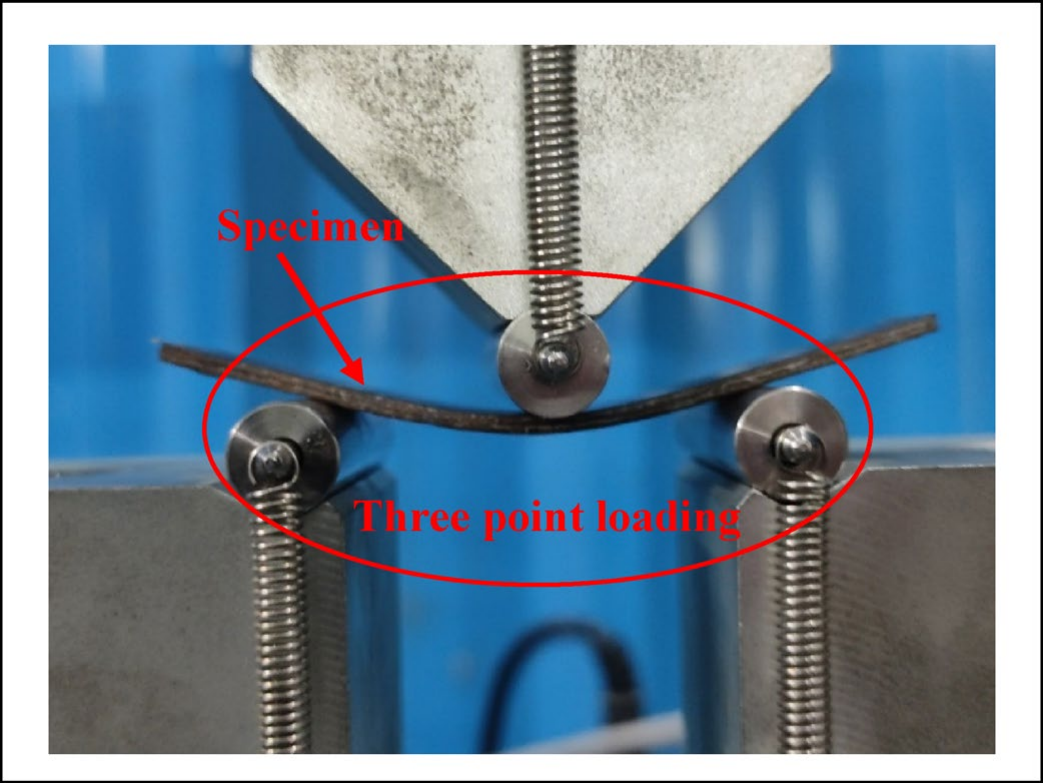
Images of the 3D specimen in bending test.

In order to explore the workability of the developed biocomposite for structural applications, the developed composites are characterized for the different physical and mechanical properties namely density, hardness, compressive strength, flexural strength, tensile strength, impact strength and wear. The density of the composite is evaluated by using the ASTM D792 standard test for density of biocomposites whereas, the hardness of the composites is evaluated by following the test standard ASTM D785 for composite using a hardness tester. The compressive strength determines the strength of a material under an external compressive load. The evaluations of the compressive strength important for structural applications. It is evaluated according to the standard ASTM D695 for compressive strength by using a universal testing machine (Tinius Olsen H50KL) as shown in Fig. 6. The tensile strength of the composite is evaluated as per the ASTM D638 test standard for composites as shown in Fig. 7. The flexural strength, also known as bending strength, gives the strength of a material under a bending load. It is evaluated as per the ASTM D790 standard test for flexural loading as shown in Fig. 8. The impact strength of the composite gives the strength of the material under a sudden loading and is an important consideration for the composite in structural applications. It is evaluated as per the ASTM 6110 test standard for impact strength. The wear performance of the biocomposites is estimated by the evaluation of specific wear rates in sliding wear test conditions by using a pin-on-disc apparatus according to the ASTM G99-17 test standard.

### Properties of the alternatives

**Fig. 9:**
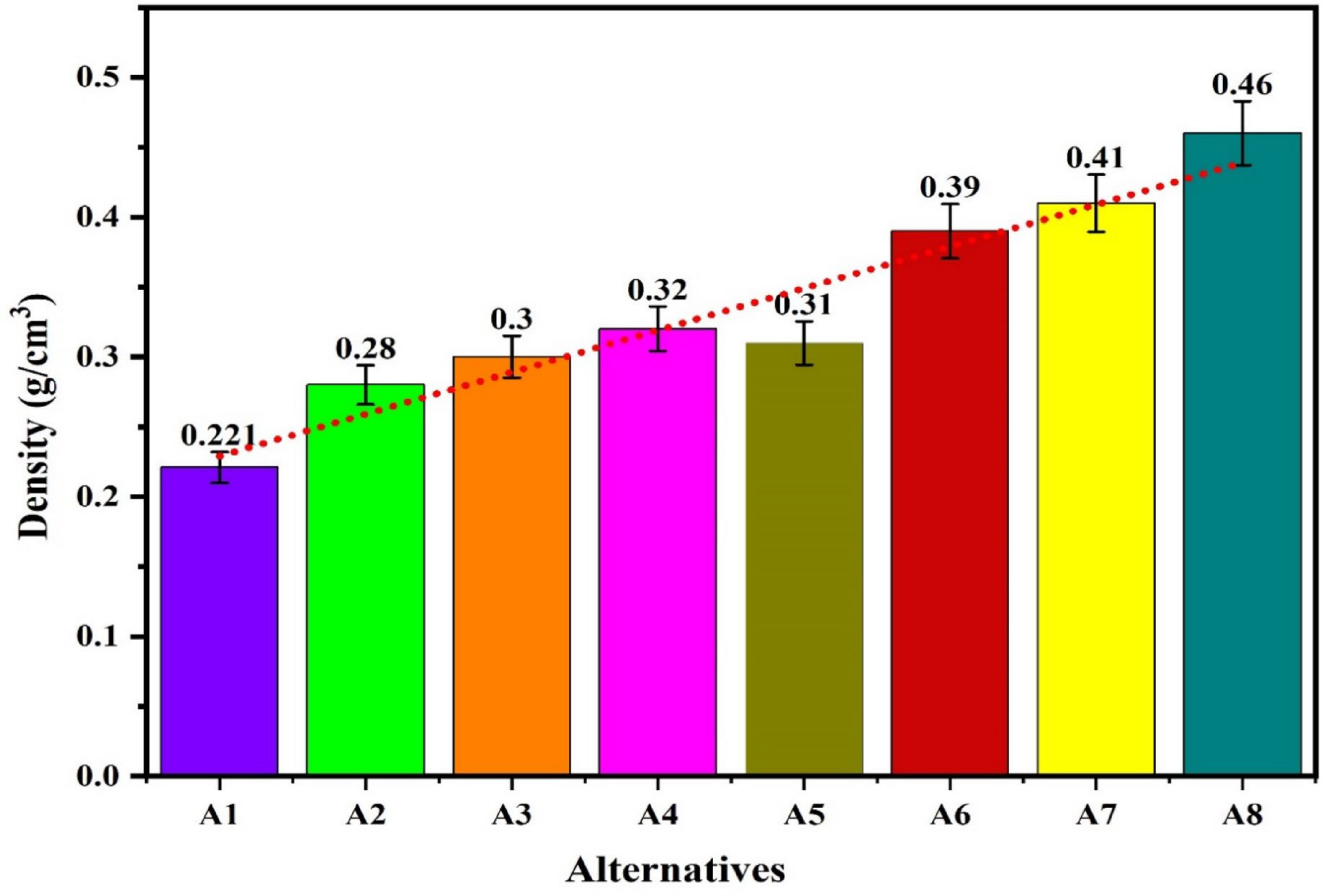
Plot for the density of the alternatives.

**Fig. 10:**
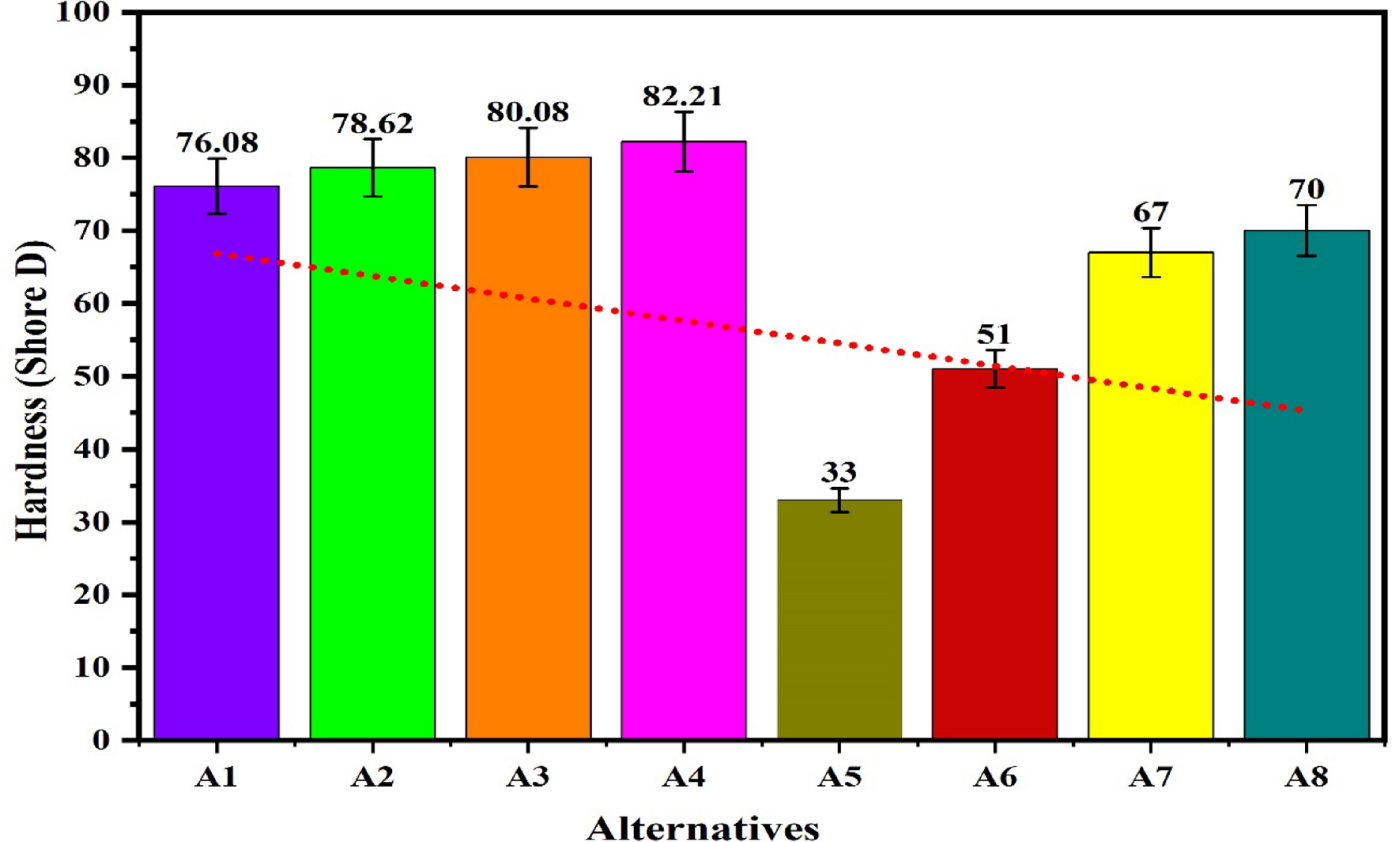
Plot for the hardness of the alternatives.

The workability of the biocomposites for structural applications has been verified by the evaluations of the density, hardness, compressive strength, flexural strength, tensile strength, impact strength and wear rate. The resulting properties are summarized in Table 2^92,93^. The plot for the density of the alternatives given in the Fig. 9 shows that the density of the alternatives is varies from 0.221 to 0.46 g/cm^3^ which indicates that the developed composites are light weight making it favourable for structural applications. The lower density for the alternatives A1 and A2 has indicated a high strength-to-weight ratio while the higher density for the alternatives A7 and A8 is due to the high compactness. The hybrid biofillers, i.e. orange and banana peel powder reinforced alternative have demonstrated a higher densities as compared to the composites having banana peel powder as reinforcement. The density of the composite increases with fractions of biofillers however, an invariability in the behaviour with compositions is observed. This invariability in the density is due to the agglomeration of the fillers and creations of the voids. Moreover, the change in the fillers morphologies like shape size and structure changes the density in an irregular manner^94^. The plot for the hardness of the alternatives in Fig. 10 shows that the hardness of the composites varies linearly with the loading fraction of the biofillers. The good binding strength between the matrix and biofiller along with uniform distribution of biofillers provides the rigidity and an effective load bearing area therefore the resistance to indentation^95^. The composites reinforced with 20 wt.% of banana peel powder has demonstrated an optimal hardness of 82.21 (shore D), whereas the composite having 5 wt.% of hybrid biofillers of orange and banana peel powder has demonstrated a minimum hardness of 33 (shore D). This significant variations for the harness of the composite is attributed to the variation in the dispersion of fillers and interfacial bonding between the matrix and filler^63^. The higher hardness i.e. > 80 (shore D) for the alternative has suggested an excellent resistance to indentation and deformation of the surface. The higher porosity and weaker bonding along with the softness of the matrix are the possible cause for the minimum hardness of the alternative A5. It is observed that for the given loading fraction of reinforcements, the banana peel powder reinforced composites have demonstrated a higher hardness as compared to the hybrid biofillers reinforced composites.

**Table 2:** Resulted properties of the alternatives.

Alternative	Density (g/cm^3^)	Hardness (shore D)	Compressive strength (MPa)	Flexural strength (MPa)	Tensile strength (MPa)	Impact strength (KJ/m^2^)	Specific wear (mm^3^/N-m)
A1	0.221	76.08	60.10	74.10	41.30	15,000	0.00114
A2	0.28	78.62	65.78	63.80	44.40	4000	0.00080
A3	0.30	80.08	70.16	58.50	36.60	10,000	0.00057
A4	0.32	82.21	68.81	47.20	32.80	20,000	0.00066
A5	0.31	33	70.25	42.1	21.2	10,000	0.00120
A6	0.39	51	39.20	39.1	15.4	15,000	0.00114
A7	0.41	67	43.92	36.8	12.9	21,000	0.00080
A8	0.46	70	51.49	44.2	11.9	18,000	0.00071

**Fig. 11:**
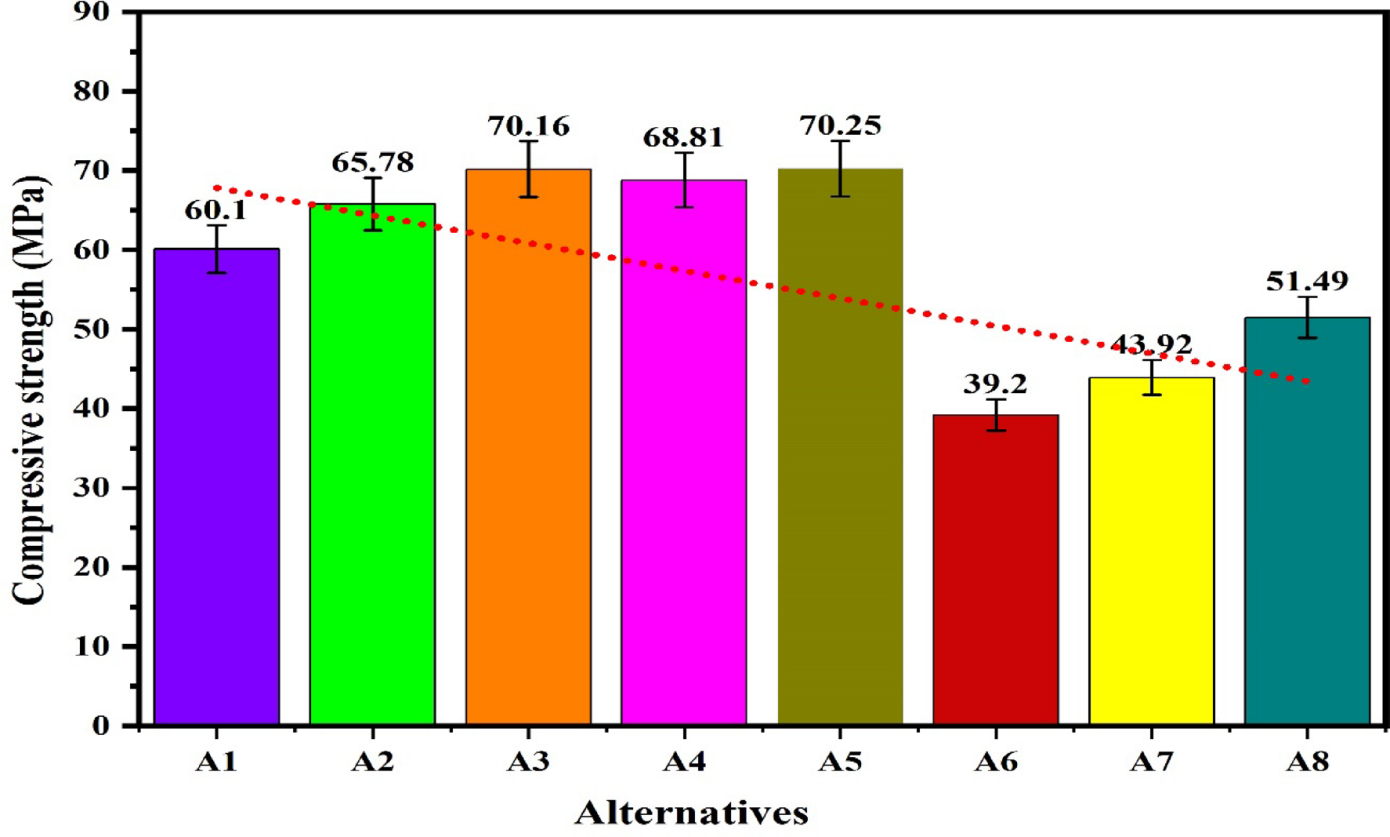
Plot for the compressive strength of the alternatives.

**Fig. 12:**
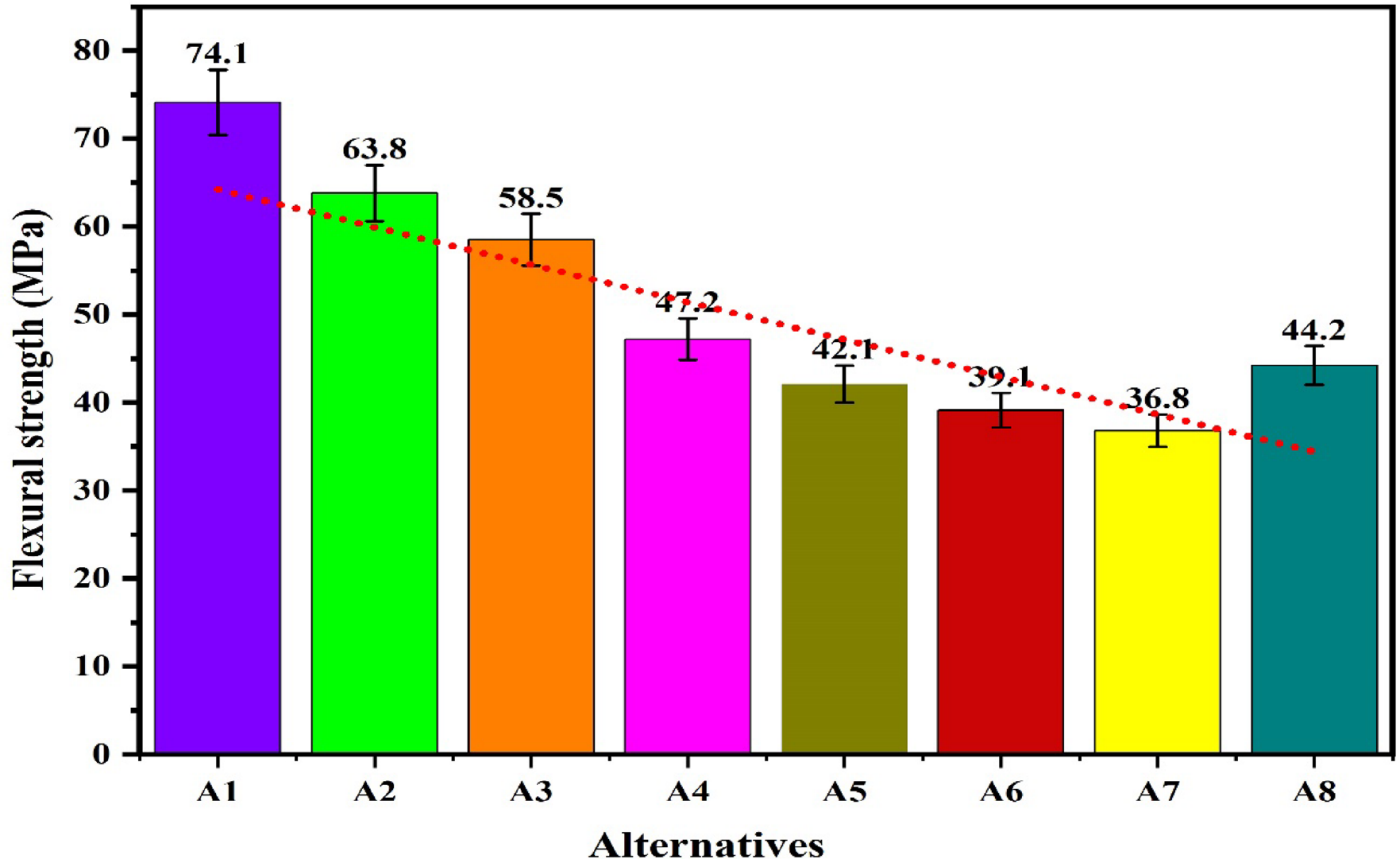
Plot for the flexural strength of the alternatives.

**Fig. 13:**
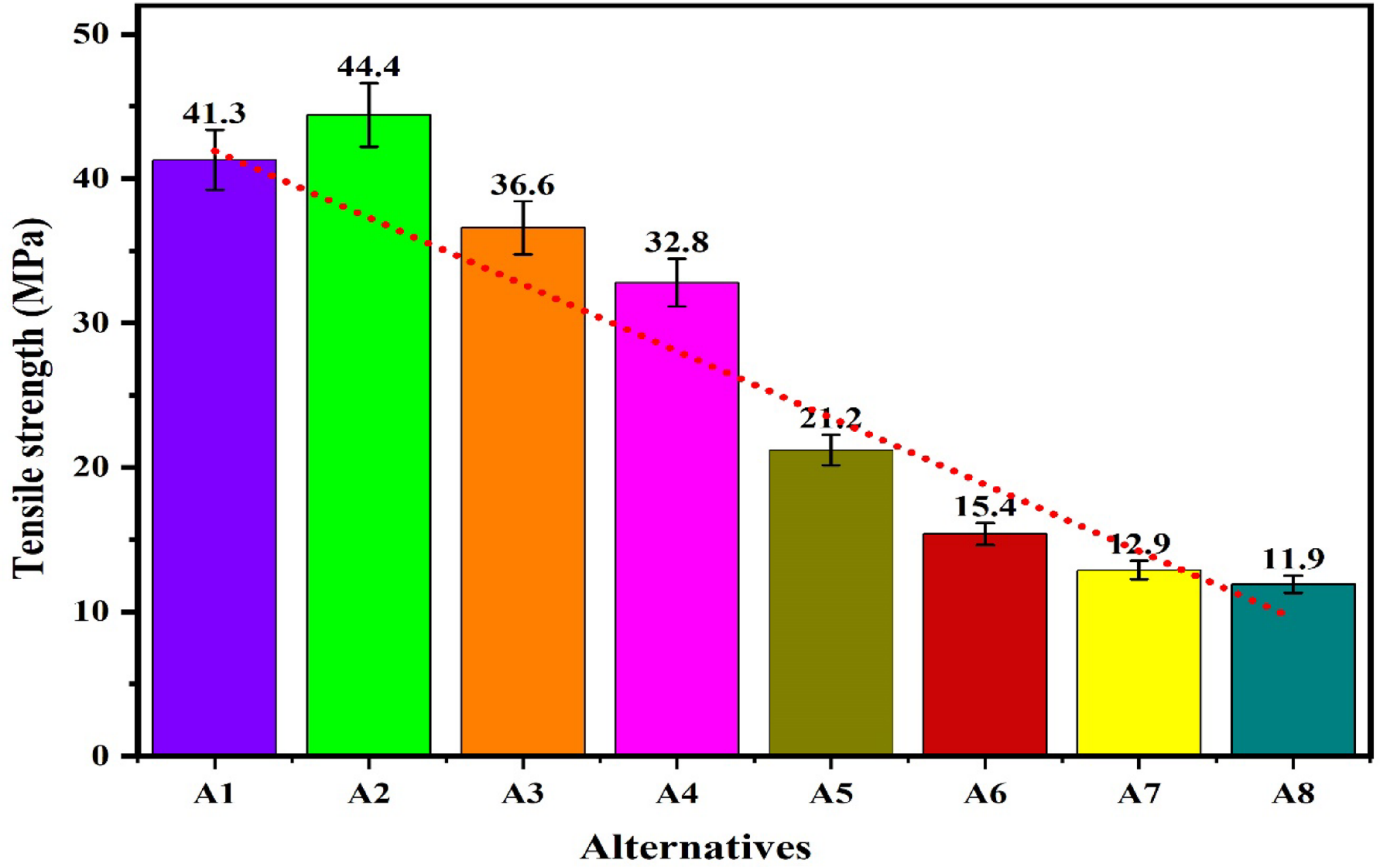
Plot for the tensile strength of the alternatives.

**Fig. 14:**
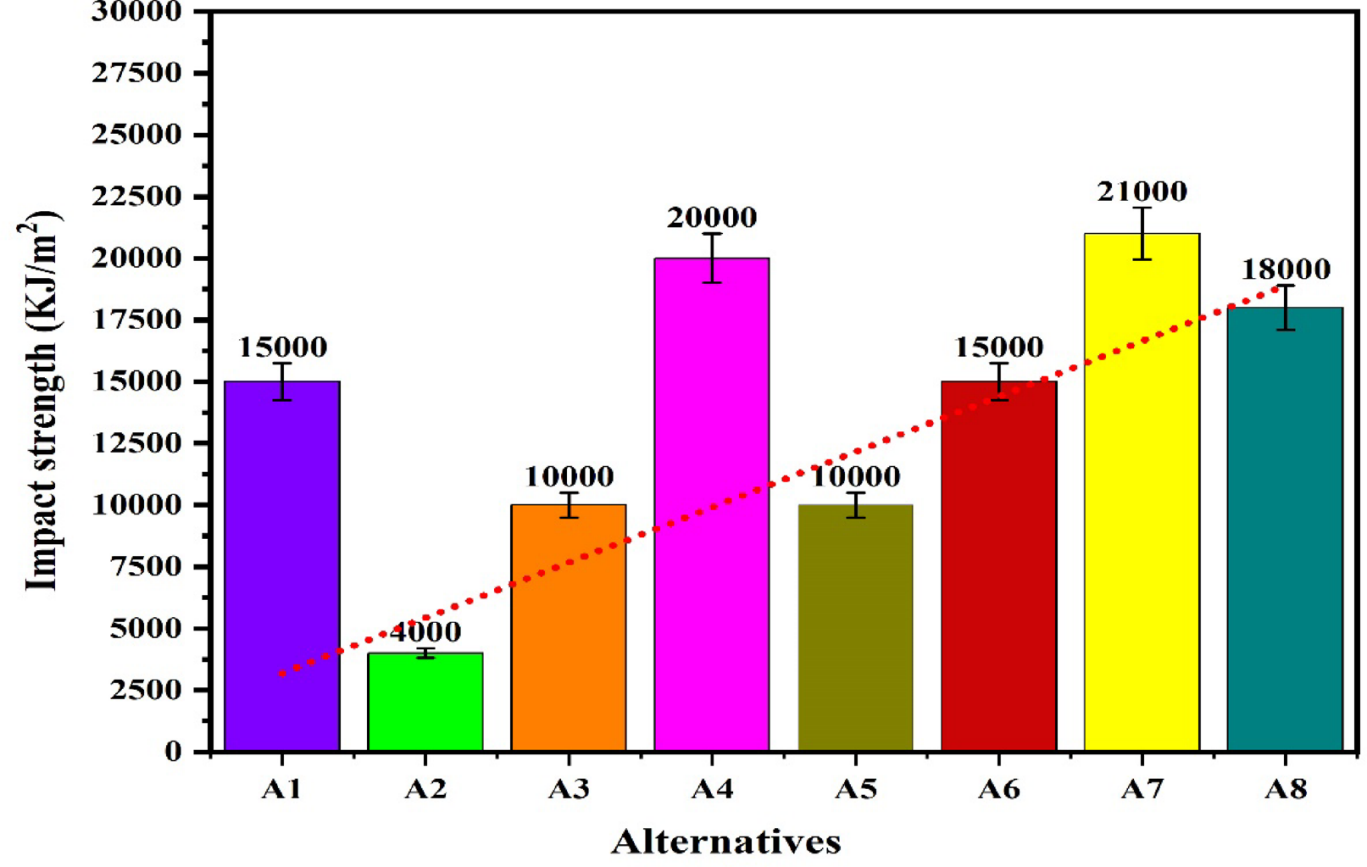
Plot for the impact strength of the alternatives.

**Fig. 15:**
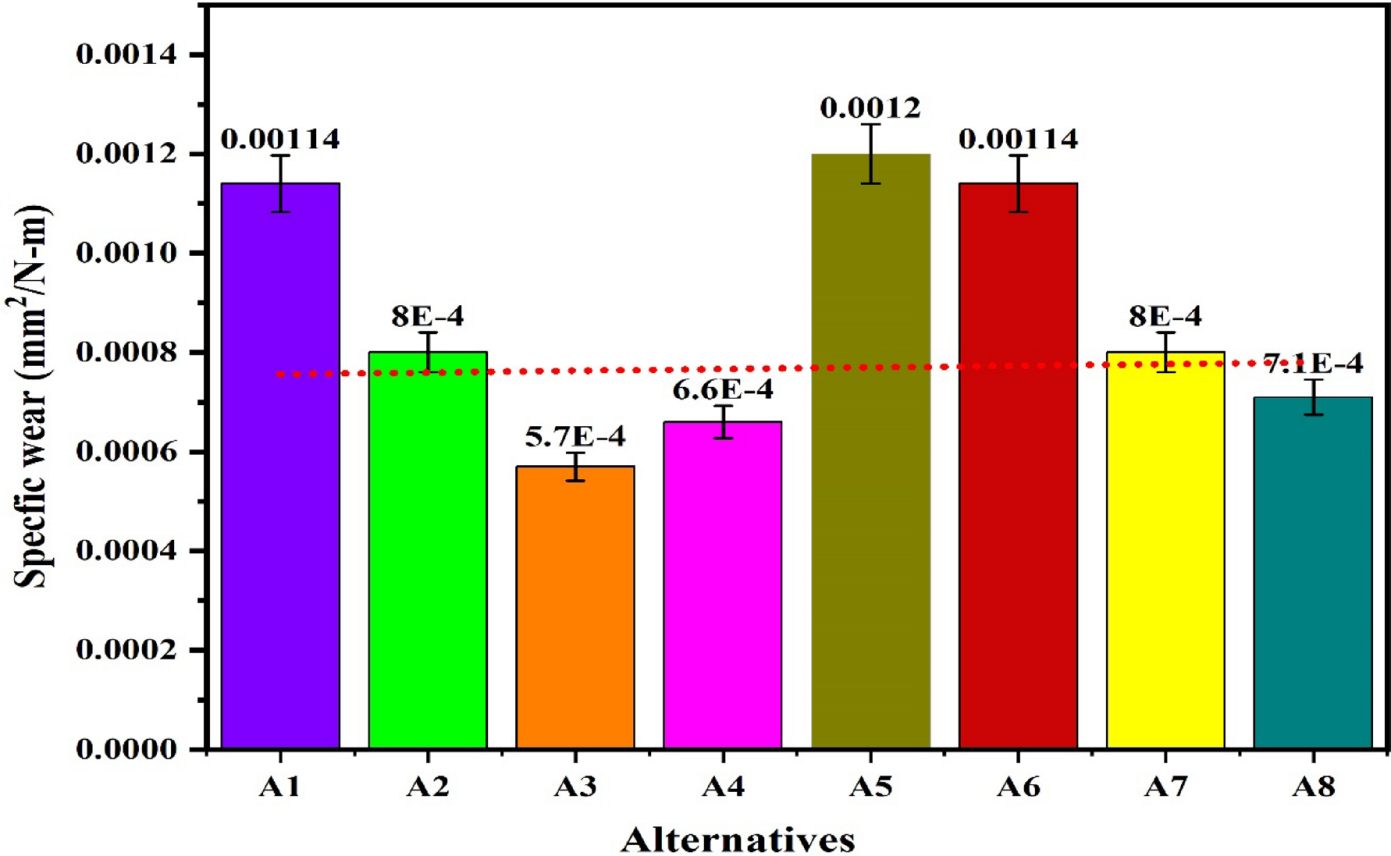
Plot for the specific wear rate of the alternatives.

The plot for the compressive strength of the alternatives in Fig. 11 shows that under the loading fraction of biofillers as BPP, the compressive strength of the samples increases with biofillers and obtains a maximum value of 70.16 MPa for the alternative having 15 wt.% of the biofillers then decreases for the composite having 20 wt.% of biofillers. The increase in the capacity to stress transfer, packing density and interfacial adhesion has increases the compressive strength with fillers loading up to 15 wt.% however further loading of fillers has resulted in the poor dispersion, agglomeration and formations of voids acting as a stress concentrators and reduces the compressive strength ^96^. A similar observation for the hybrid biofilles composites has demonstrated a decreasing and increasing trend with fillers loadings for the hybrid biofillers reinforced composite resulting in a minimum compressive strength of 39.2 MPa for the composite having 10 wt.% of biofillers. The high compressive strength of the alternative A3 and A5 indicates the capacity of the material to withstand the compressive load without failure and suggested their applications in load bearing and structural applications. The plot for the flexural strength shown in Fig. 12 indicates that the flexural strength of the biocomposites decreases with the fraction of reinforcement for the BPP reinforced composites. The poor interfacial bonding due to the hydrophilic nature of BPP and hydrophobicity of PLA decreases the matrix continuity acting as stress concentrator and reduces the load transfer capacity as a result the matrix become brittle which decreases the flexural strength with loading fraction of BPP ^97^. The observations for the flexural strength for the hybrid biofillers reinforced composites the flexural strength of the composite decreases with reinforcement however due to the irregularity in the behaviour, the flexural strength of the composite increases for the composites having an optimal fraction of hybrid biofillers. The synergic interaction between the hybrid biofillers having different morphologies and surface characteristics provides the uniform dispersion and enhances the adhesion with the PLA matrix. The resulted microstructure favours the efficient load transfer and a good balance between the stiffness and toughness which increases the flexural strength at optimal fraction of hybrid bio fillers^98^. The maximum and minimum flexural strength are found to be 74.1 MPa and 36.8 MPa, respectively. The alternative A1 has demonstrated the highest flexural strength which indicates an excellent resistance to bending and matrix-filler adhesion whereas the insufficient interfacial bonding an agglomerations of the fillers has resulted to minimum flexural strength for the alternative A7. The tensile strength of the composite are found in the ranges of 11.9 MPa and 44.4 MPa. The alternative A2 has denoted an optimal tensile strength which reflects the effectiveness of stress transfer between the matrix and the biofiller, as well as the composite’s overall structural integrity. The tensile is influenced by filler aspect ratio and stress transfer efficiency ^99^. The lower tensile strength for the alternative A8 and A7 suggests weak bonding or higher void content. The plot for the tensile strength in Fig. 13 shows that the tensile strength of the composite increases with the reinforcement of 10 wt.% of banana peel powder then decreases with further reinforcement of biofillers whereas the tensile strength decreases invariably with reinforcement of hybrid biofillers. The tensile strength of the PLA-based composite increases improves with the addition of 10 wt.% of BPP) with 90 wt.% of PLA due to the enhancement in the load transfer between the matrix and uniformly distributed rigid biofiller particles. At this intermediate concentration, the BPP serves as a strong reinforcement by limiting polymer chain movement and increasing stiffness, all without greatly disturbing the continuity of the matrix. The effective adhesion between the filler and the matrix at this stage ensures efficient stress transfer, resulting in optimal tensile strength ^100^. However, as the BPP content continues to rise, the tensile strength decreases due to the clumping of fillers, inadequate dispersion, and weak bonding at the interface. The increased filler loading leads to the creation of voids and areas of stress concentration which impair load transfer and encourage early crack formation when under tension ^101^. Additionally, the polymer matrix becomes less continuous, diminishing its capacity to handle tensile deformation. The poor interfacial adhesion between the hydrophilic biofillers of BPP and OPP, and hydrophobic PLA matrix along with the agglomerations and formations of voids promotes the stress concentration and increases the brittleness as a result reduces the tensile strength with fractions of biofillers. The impact strength of the composite increases then decreases with reinforcement resulting in an irregular trend as shown in Fig. 14. The maximum impact strength is found to be 21,000 kJ/m^2^ for the alternative A7 having 15 wt.% of hybrid biofillers of orange and banana peel powder. The higher impact strength of the alternatives A7 and A4 attributed to the effective dissipation of the energy through the crack deflection or micro-debonding mechanisms making the materials suitable for shock resistance ^102^. The impact strength varies considerably as compared to the flexural and tensile strength because it is defect-sensitive, rate-dependent, and governed by multiple competing failure modes*.* It is observed that under a similar loading fraction of reinforcement, the hybrid biofillers reinforced composite performed better as compared to the composite having a single type of biofiller. The results of the specific wear rate plotted in Fig. 15 shows that the wear of the banana peel powder reinforced biocomposites decreases with reinforcement attaining a minimum value of 0.00057 mm^2^/N-m, then increases to 0.00066 mm^2^/N-m, whereas for the hybrid biofillers reinforced composites, the wear of the samples decreases with the reinforcements. The specific wear rate of the composites are in the ranges of 0.00057–0.00114 (mm^2^/N-m) which is comparable to the work where the incorporations of 5 wt.% of silicon particle in 3D printed PLA has demonstrated a specific wear rate of 0.0002 mm^3^/N-m ^103^. The alternative A3 has obtained a minimum wear rate therefore denotes an excellent tribological stability. The higher wear rate for the alternative A5 and A1 indicates a higher degradation under frictional contact. This irregular behaviour of the wear response is attributed to the competing influence of biofillers reinforcement and interfacial weakness. The incorporation of biofillers increases the strength and hardness while the factors such as agglomeration, non-uniform dispersion and poor interfacial adhesion lead to the inconsistency in the wear performance with filler loadings^104^. Therefore, an irregularity in the wear response for the developed biocomposites is observed. The comparative analysis of the properties has observed that the workability of the composite varies irregularly with composition. Therefore, requires an effective mathematical model for ranking the alternatives and identifying a suitable composition.

### Mathematical formulations

This section describes the mathematical tool which is applied for the current study.

### Triangular fuzzy number (TFN)

A TFN ã can be illustrated through a triplet $({a}^{l}, {a}^{m}, {a}^{u})$. The membership function ${\mu }_{\widetilde{a}} (x)$ is defined as:

$${\mu }_{\widetilde{a}}\left(x\right)=\left\{\begin{array}{c}0,x<{a}^{l}\\ \frac{x-{a}^{l}}{{a}^{m }-{a}^{l}}, {a}^{l}\le x\le {a}^{m}\\ \frac{x- {a}^{u}}{{a}^{m}-{a}^{u}}, {a}^{l}\le x\le {a}^{m}\\ o, x\ge {a}^{u}\end{array}\right.$$

where 0 < ${a}^{l}\le {a}^{m }\le {a}^{u}$, ${a}^{l}$ and ${a}^{u}$ denote the lowest and highest values of the support of $\tilde{a}$ respectively and ${a}^{m}$ symbolizes the mean value.

### Basic operational laws related to TFNs

Let $\widetilde{a}$ and $\widetilde{b}$ be two TFNs. The fundamental expressions are given as follows:

(i) $$\widetilde{a}\oplus \widetilde{b} =$$ ($${a}^{l}$$,$${a}^{m}$$, $${a}^{u}$$)$$\oplus$$ ($${b}^{l}$$, $${b}^{m}$$, $${b}^{u}$$) = ($${a}^{l}$$+$${b}^{l}$$, $${a}^{m}$$+$${b}^{m}$$,$${a}^{u}$$+$${b}^{u}$$)
(ii) $${\tilde{a} } \otimes \widetilde{b}$$ =$$( {a}^{l}, {a}^{m}, {a}^{u}) \otimes ( {b}^{l}, {b}^{m}, {b}^{u}$$) = ($${a}^{l}{b}^{l}$$, $${a}^{m}{b}^{m}$$, $${a}^{u}{b}^{u}$$)
(iii) $$\lambda \otimes$$ ã = λ $$\otimes$$ ($${a}^{l}$$,$${a}^{m}$$,$${a}^{u}$$)$$=(\lambda {a}^{l}, \lambda {a}^{m}, \lambda {a}^{u}), \lambda >0$$
(iv) Defuzzification: $$Center of Area (COA)= \frac{{a}^{l}+{a}^{m}+ {a}^{u}}{3}$$
(v) $${\widetilde{a}}^{-1}$$ = $${\left({a}^{l}, {a}^{m},{a}^{m}\right)}^{-1}$$= $$\left(\frac{1}{{a}^{n}},\frac{1}{{a}^{m}},\frac{1}{{a}^{l}}\right)$$

### Bonferroni mean operator (BMO)

Bonferroni mean operator was presented by Bonferroni ^105^, that is fundamentally a mean type AO. It can provide an aggregation lying among the min, max operators and the logical “or” and “and” operators, it can be defined as:

Let $p,q\ge 0$ be the parameters and ${a}_{i }(i=\text{1,2},\dots \dots .n)$ be a collection of non- negative real numbers. Then the aggregation function

$${{BM}^{p,q}({a}_{i, }{a}_{2},\dots ,a}_{n}){=\left(\frac{1}{n(n-1)}\sum_{\begin{array}{c}i,j=1\\ i\ne j\end{array}}^{n}{a}_{i}^{p}{a}_{j}^{p}\right)}^{\frac{1}{p+q}}$$

It is a BM operator.

### Weighted Bonferroni mean (WBM) operator

Let $p,q\ge$ 0 and ${a}_{i}$ be the collection of non-negative numbers. ${w}_{i }(i={1,2},....n)$ is the weight vector of ${a}_{i}$ where ${w}_{i}$ denotes the degree of importance of ${a}_{i}$, satisfying $0{<w}_{i } < 1, \left(i={1,2},\dots \dots .n\right),$ and $\sum_{i=1}^{n}{w}_{i}=1.$ Then WBM operator is defined as follows.

$${WBM}^{p,q} ({a}_{1,\dots }{a}_{n})={\left(\frac{1}{n(n-1)}\sum_{\begin{array}{c}i,j=1\\ i\ne j\end{array}}^{n}{{(w}_{i}{a}_{i})}^{p}{{(w}_{j}{a}_{i})}^{q}\right)}^{\frac{1}{p+q}}$$

### Triangular fuzzy Bonferroni mean operator (TFBMO)

The BM operators can be prolonged to house the situation where the input arguments are TFNs. The explanation of the TFBM operator is as follows:

Let ${\widetilde{a}}_{i}$ = (${a}_{i}^{l}$,${a}_{i}^{m}$,${a}_{i}^{u}$) (*i* = 1,2,……, n) be a set of TFN and let *p*,* q* > *0.*

Then, ${TFBM}^{p.q}$ is defined as

$$\begin{aligned} & TFBM^{p.q} \left( {a_{1} ,{ }a_{2} , \ldots { }, a_{n} } \right) \\ & = \left[ {\begin{array}{*{20}c} {\left( {\frac{1}{{n\left( {n - 1} \right)}}\mathop \sum \limits_{{\begin{array}{*{20}c} {i,j = 1} \\ {i \ne j} \\ \end{array} }}^{n} (a_{i}^{l} )^{p} (a_{j}^{l} )^{q} )^{{\frac{1}{{\left( {p + q} \right)}}}} } \right),} \\ {\left( {\frac{1}{{n\left( {n - 1} \right)}}\mathop \sum \limits_{{\begin{array}{*{20}c} {i,j = 1} \\ {i \ne j} \\ \end{array} }}^{n} (a_{i}^{m} )^{p} (a_{j}^{m} )^{q} )^{{\frac{1}{{\left( {p + q} \right)}}}} } \right),} \\ {\left( {\frac{1}{{n\left( {n - 1} \right)}}\mathop \sum \limits_{{\begin{array}{*{20}c} {i,j = 1} \\ {i \ne j} \\ \end{array} }}^{n} (a_{i}^{n} )^{p} (a_{j}^{n} )^{q} )^{{\frac{1}{{\left( {p + q} \right)}}}} } \right)} \\ \end{array} } \right] \\ \end{aligned}$$

The expression and terms associated with TFBM operator has been illustrated below:

#### Theorem 3.4.1

Let ${\widetilde{a}}_{i}$ = [${a}_{i}^{l}$, ${a}_{i}^{m}$,${a}_{i}^{u}$] (*i* *= *1,2,…,* n*) be the collection of TFNs and $p,q\ge 0,$ then the outcomes collected from the definition 3.4 is still a TFN.

#### Property 1:

(Idempotency) Let ${\widetilde{a}}_{i}$ = [${a}_{i}^{l}$, ${a}_{i}^{m}$,${a}_{i}^{u}$] *(i* = *1,2,……, n)* be a set of TFNs. If all $\widetilde{{a}_{j}}\left(\widetilde{{a}_{j}}=\left[{a}_{j}^{l},{a}_{j}^{m},{a}_{j}^{u}\right]\right)$ are equal that is $\widetilde{{a}_{j}}\left(\widetilde{{a}_{j}}=\left[{a}_{j}^{l},{a}_{j}^{m},{a}_{j}^{u}\right]\right)= \widetilde{a}\left(\widetilde{a }=\left[{a}^{l}, {a}^{m},{a}^{u}\right]\right)$ for all $j,$ then,

$${TFBM}^{p.q} {\widetilde{a}}_{1},{\widetilde{a}}_{2}, {\widetilde{a}}_{n}, =\widetilde{a}$$

#### Property 2:

(Boundedness)Let ${\widetilde{a}}_{i}$ = [${a}_{i}^{l}$,${a}_{i}^{m}$,${a}_{i}^{u}$] *(i* = *1,2,……, n)* be a set of TFNs and let.

$${\widetilde{a}}^{- }=\text{min}\widetilde{{a}_{j}}, {\widetilde{a}}^{+ }=\text{max}\widetilde{{a}_{j}}$$

Then

$${\widetilde{a}}^{- }\le {TFBM}^{p,q}\left({\widetilde{a}}_{1} ,{\widetilde{a}}_{2} ,\dots .., {\widetilde{a}}_{n}\right)\le {\widetilde{a}}^{+}$$

#### Property 3:

(Monotonocity)Let ${\widetilde{a}}_{i}$ = [${a}_{i}^{l}$,${a}_{i}^{m}$,${a}_{i}^{u}$] *(i* = *1,2,……, n)* and $\widetilde{{a}_{j}}^{\prime}$= [${a{\prime}}_{i}^{l}$, ${a{\prime}}_{i}^{m}$,${a{\prime}}_{i}^{u}$]

*(i* = *1,2,……, n)* be two sets of TFNs, if ${\widetilde{a}}_{j}\le {{\widetilde{a}}^{\prime}}_{j},$ for all $j,$ then.

$${TFBM}^{p,q}{\widetilde{a}}_{1},{\widetilde{a}}_{2},\dots \dots .{\widetilde{a}}_{n}$$()$$\le {TFBM}^{p.q}\left({{\widetilde{a}}^{{{\prime}}}}_{1}, {{\widetilde{a}}^{{{\prime}}}}_{2},\ldots {{\widetilde{a}}^{{{\prime}}}}_{n}\right)$$

#### Property 4:

(Commutativity)Let ${\widetilde{a}}_{i}$ = [${a}_{i}^{l}$,${a}_{i}^{m}$,${a}_{i}^{u}$]*( i* = *1,2,……, n)* and $\widetilde{{a}_{j}}^{\prime} =$ [${a{\prime}}_{i}^{l}$, ${a{\prime}}_{i}^{m}$,${a{\prime}}_{i}^{u}$]

*(i* = *1,2,……, n)* be two sets of TFN, where ${\widetilde{{a}_{i}}}^{\prime}=\left[{{\widetilde{{a}_{i}}}^{\prime I}}, {{\widetilde{{a}_{i}}}^{\prime m}} , {{\widetilde{{a}_{i}}}^{\prime u}}\right](i={1,2},\ldots .n)$ is any permutation of ${\widetilde{a}}_{i}$ = [${a}_{i}^{l}$, ${a}_{i}^{m}$,${a}_{i}^{u}$] *( i* = *1,2,……, n),* then ${TFBM}^{P.q}\left({\widetilde{a}}_{1} , {\widetilde{a}}_{2} ,\dots \dots ..{\widetilde{a}}_{n}\right)= {\widetilde{a}}_{1}^{\prime}, {\widetilde{a}}_{2}^{\prime}, ,\dots \dots ..{\widetilde{a}}_{n}^{\prime},$.

Now, we discuss some special cases of the TFBM with respect to the parameters $p$ and $q:$

**Case 1** If $q\to 0,$ then from the TFBM, it yields $$\underset{q\to 0}{\text{lim}}{TFBM}^{p.q}\left({\widetilde{a}}_{1},{\widetilde{a}}_{1},\dots .{\widetilde{a}}_{n}\right)\underset{q\to 0}{\text{lim}}{\left(\frac{1}{n(n-1)}\sum_{\begin{array}{c}i,j=1\\ i\ne j\end{array}}^{n}{\widetilde{{a}_{i}}}^{p}{\widetilde{{a}_{i}}}^{q}\right)}^{\frac{1}{p+q}} ={\left(\frac{1}{n}\sum_{i=1}^{n}{\widetilde{{a}_{i}}}^{p}\right)}^\frac{1}{p}$$=${TFBM}^{p.o}\left({\widetilde{a}}_{1},{\widetilde{a}}_{2},\dots .{\widetilde{a}}_{n}\right)$, is a TFGM operator.

**Case 2** If $p=2$ and $q\to 0,$ them by the TFBM, we have ${TFBM}^{\text{2,0}}\left({\widetilde{a}}_{1}, {\widetilde{a}}_{2},\dots ..{\widetilde{a}}_{n}\right)={\left(\frac{1}{n}\sum_{i=1}^{n}{\widetilde{a}}_{{i}^{2}}\right)}^\frac{1}{2}$ whichis called the TFSM operator.

**Case 3** If $p=1$ and $q\to 0$, then the TFBM downgraded to the TFM operator.${TFBM}^{\text{1,0}} ({\widetilde{a}}_{1}$ ,${\widetilde{a}}_{2}\dots \dots .,{\widetilde{a}}_{n})$)$=\frac{1}{n}\sum_{i=1}^{n}{\widetilde{a}}_{i}$, is a TFM operator.

**Case 4** If $p=1$ and $q=1$, then the TFBM, we have.

$${TFBM}^{\text{1,1}}$$($$\widetilde{{a}_{i}}$$, $${\widetilde{a}}_{2}$$,……..$${\widetilde{a}}_{n})= {\left(\frac{1}{n\left(n-1\right)}\sum_{\begin{array}{c}i,j=1\\ i\ne j\end{array}}^{n}\widetilde{{a}_{i}}\widetilde{{a}_{j}}\right)}^\frac{1}{z}$$

Which is called the TFISM operator.

### Triangular fuzzy weighted Bonferroni mean operator (TFWBMO)

Let ${\widetilde{a}}_{i}$ = [${a}_{i}^{l}$, ${a}_{i}^{m}$,${a}_{i}^{u}$] *( i* = *1,2,……, n)*be a set of sets of TFNs and $p,q>0, w= {\left({w}_{1},{w}_{2},\dots \dots {w}_{n}\right)}^{T}$ is the weight vector of ${\widetilde{a}}_{i}$ = [${a}_{i}^{l}$, ${a}_{i}^{m}$,${a}_{i}^{u}$] *( i* = *1,2,……, n),* where ${w}_{i}$ indicates the importance degree of ${\widetilde{a}}_{i}$, satisfying ${w}_{i}>0 \left(i=1, 2,\dots \dots ..n\right)$ and $\sum_{i=1}^{n}{w}_{i}=1$. Thus, the TFWBM can be defined as

$${TFWBM}_{w}^{p,q} ({\widetilde{a}}_{1}, {\widetilde{a}}_{2}, \dots \dots \dots {\widetilde{a}}_{n})={\left({\frac{1}{n\left(n-1\right)}\sum_{\begin{array}{c}i,j=1\\ i\ne j\end{array}}^{n}{({w}_{i}{\widetilde{a}}_{i})}^{p}{ ({w}_{j}{\widetilde{a}}_{j})}^{q})}^{\frac{1}{(p+q)}}\right)}^{\frac{1}{p+q}}$$

### Proposed aggregation operator based mathematical model

In this section, the details of the Triangular Fuzzy Weighted Bonferroni Mean Operator (TWWBMO) based CRITIC and COPRAS method for the ranking of the alternatives are provided.

### Proposed TFWBMAO based fuzzy CRITIC

CRITIC was proposed by Diakoulaki, Mavrotas, and Papayannakis in 1995 and is a popular method for criteria weighting which eliminates the subjective bias in determining the weights ^106^. The criteria weights depend on the data in the decision matrix. The technique maintains the conflict among the criteria and provides unique information ^107^. The method is helpful in the separation of subjective bias through weight determination and captures the critical aspects of the criteria ^108^. The method requires the prior conversion of linguistic data into numerical data; also, the weight is affected due to the error in the decision matrix ^109^. The method is sensitive to the data, and the errors in the decision matrix can influence the criteria weight. The algorithm of the proposed TFWBMAO based Fuzzy CRITIC method is given as follows:

Suppose, here *l* DMs symbolized by ${DM}_{r}, (r = \text{1,2},3........l)$, $m$ alternatives ${A}_{i} (i = \text{1,2},3.....m)$ and $n$ evaluation criteria ${C}_{j} (j= \text{1,2},3.....,n)$*.* The algorithm of the anticipated TFWBMAO-based CRITIC is given below:

Step 1: Construct the decision matrix.

The decision matrix involving the alternatives and attributes is given in Eq. (1)

1 $$\left[\begin{array}{cccccc}{r}_{11}& \dots & {r}_{1j}& {r}_{1,j+1}& \dots & {r}_{1n}\\ \vdots & \ddots & \vdots & \vdots & \ddots & \vdots \\ {r}_{ii}& \cdots & {r}_{ij}& {r}_{i,j+1}& \cdots & {r}_{in}\\ \vdots & \ddots & \vdots & \vdots & \ddots & \vdots \\ {r}_{m1}& \cdots & {r}_{mj}& {r}_{j,m+1}& \cdots & {r}_{mn}\end{array}\right]$$

where *i* = 1, 2, …, *m*; *j* = 1, 2, …, *n* and $\left\{{A}_{1 },{A}_{1 },\cdots \cdots ,{A}_{m}\right\}$ and ${r}_{ij}$ I for is the element of the decision matrix for *i*_*th*_ alternative and *j*_*th*_ attribute.

Step 2: Normalize the decision matrix.

In order to normalize the positive (benefit) and negative (cost) attributes of the decision matrix, Eqs. 2 and 3, respectively.

2 $${x}_{ij}=\frac{{r}_{ij}-{r}_{i}^{-}}{{r}_{i}^{+}-{r}_{i}^{-}}i=1, 2,, \dots ,m; j=1, 2, \dots ,n$$

3 $${x}_{ij}=\frac{{r}_{ij}-{r}_{i}^{+}}{{r}_{i}^{+}-{r}_{i}^{+}}i=1, 2, \dots , m; j=1, 2, \dots ,n$$

where *x*_*ij*_ represents the normalized values of the decision matrix for *i*_th_ alternative with respect to *j*_*th*_ attribute and ${r}_{i}^{+}=\mathit{max}\left({r}_{1},{r}_{1},\dots .,{r}_{m}\right), {r}_{i}^{\_}=min\left({r}_{1},{r}_{1},\dots .,{r}_{m}\right)$.

Step 3: Find the correlation coefficients.

The correlation coefficient ${\rho }_{jk}$ between attributes is determined by Eq. 4.

4 $${\rho }_{jk=\raisebox{1ex}{$\sum_{i=1}^{m}\left({x}_{ij}-{\overline{x} }_{j}\right)\left({x}_{ik}-{\overline{x} }_{k}\right)$}\!\left/ \!\raisebox{-1ex}{$\sqrt{\sum_{i=1}^{m}{\left({x}_{ij}-{\overline{x} }_{j}\right)}^{2}\sum_{i=1}^{m}{\left({x}_{ik}-{\overline{x} }_{k}\right)}^{2}}$}\right.}$$

where ${\overline{x} }_{j}$ and ${\overline{x} }_{k}$ are the means of *j*_*th*_ and *k*_*th*_ attributes. ${\overline{x} }_{j}$ is obtained from Eq. 4 and similarly for ${\overline{x} }_{k}$ [2].

5 $${\overline{x} }_{j}=\frac{1}{n}\sum_{j=1}^{n}{x}_{ij};i=1, 2,\dots ,m$$

Step 4: Calculate the sample standard deviation.

The sample standard deviation of each attribute is calculated by Eq. 6.

6 $${\sigma }_{j}=\sqrt{\frac{1}{n-1}\sum_{j=1}^{n}{\left({x}_{ij}-{\overline{x} }_{j}\right)}^{2}, i = 1, 2, \dots ,\text{ m}}$$

Step 5: Obtain the index (${C}_{j}$).

Then the index (${C}_{j}$) is calculated using Eq. 7.

7 $${C}_{j}={\sigma }_{j}\sum_{k=1}^{n}\left(1-{\rho }_{jk}\right), j=1, 2, \dots , n$$

Step 6: Calculate the weights of attributes ${w}_{j}$.

The weights of attributes are calculated by Eq. 8.

8 $${w}_{j}=\frac{{C}_{j}}{\sum_{j=1}^{n}{C}_{j}}$$

### TFWBMO based fuzzy COPRAS

COPRAS method is first introduced by Zavadskas and Kaklauskas ^110^. CORPAS is a prominent multi-criteria decision-making technique that can effectively rank the alternatives. This method is suitable for the comparison of different criteria by selecting them into similar scales ^111^. This method depends on assessing the superiority of a single alternative over other alternatives by rating and evaluation process. It follows a systematic approach such as matrix creation, conversion in a single scale, multiplying scaled value with assigned weight, calculation of summed weighted normalized values for useful and non-useful criteria, ranking of alternatives, etc.^112^. This method can be used for various applications such as project evaluation, material selection, environmental management, etc. COPRAS method considers both the beneficial and non-beneficial and is easily applicable. The method reduces the subjectivity in the decision process. It is a versatile method that is applicable in different domains such as engineering, business, and environmental evaluation ^113^. The method has the limitation of change in the ranking with input data also the output depends on the assignment of weight to criteria that need expert opinion and can introduce some subjectivity. The implementation of triangular fuzzy numbers allows the incorporation of linguistic terms and uncertainties into the decision-making process. The algorithm of the proposed TFWBMO based fuzzy COPRAS is described below:

Step 1: Formulate the triangular fuzzy decision matrix ${\widetilde{R}}^{r}$, proposed by the DMs as follows:

9 $$\tilde{R}^{r} = \begin{array}{*{20}l} {} \hfill & {C_{1}^{r} } \hfill & {C_{2}^{r} } \hfill & \ldots \hfill & {C_{n}^{r} } \hfill \\ {A_{1}^{r} } \hfill & {\tilde{a}_{11}^{r} } \hfill & {\tilde{a}_{12}^{r} } \hfill & \ldots \hfill & {\tilde{a}_{1n}^{r} } \hfill \\ {} \hfill & \ldots \hfill & \ldots \hfill & \ldots \hfill & \ldots \hfill \\ {A_{m}^{r} } \hfill & {\tilde{a}_{m1}^{r} } \hfill & {\tilde{a}_{m1}^{r} } \hfill & \ldots \hfill & {\tilde{a}_{mn}^{r} } \hfill \\ \end{array}$$

Step 2: Find the aggregated decision matrix using TFWBM operator by allocating different weights to each DM as given:

10 $$\tilde{R}^{r} = \begin{array}{*{20}l} {} \hfill & {C_{1} } \hfill & {C_{2} } \hfill & \ldots \hfill & {C_{n} } \hfill \\ {A_{1} } \hfill & {\tilde{a}_{11} } \hfill & {\tilde{a}_{12} } \hfill & \ldots \hfill & {\tilde{a}_{1n} } \hfill \\ {} \hfill & \ldots \hfill & \ldots \hfill & \ldots \hfill & \ldots \hfill \\ {A_{m} } \hfill & {\tilde{a}_{m1} } \hfill & {\tilde{a}_{m1} } \hfill & \ldots \hfill & {\tilde{a}_{mn} } \hfill \\ \end{array}$$

Step 3: Normalization of the fuzzy decision matrix by using the Eq. 11.

11 $${\overline{R} }_{ij}=\frac{{R}_{ij}}{\sum_{i=1}^{m}{R}_{ij}};i=\text{1,2},3\dots \dots \dots m;j=\text{1,2},3\dots .n$$

Step 4: Obtain the weighted normalized decision-making matrix $\widehat{R}$ by multiplying the weights to the normalized decision matrix by using the Eq. 12.

12 $${\widehat{R}}_{ij}={\overline{R} }_{ij}.{w}_{j}=\text{1,2},3\dots \dots \dots m;j=\text{1,2},3\dots .n$$

Step 5: Compute the sum of criteria for that the optimum value is most preferable for each alternative by using the Eq. 13.

13 $$\tilde{P}_{j} = \sum\limits_{j = 1}^{m} {\tilde{x}_{ij} }$$

Step 6: Calculate the sum of criteria for that the minimum value is most preferable for each alternative by using the Eq. 14.

14 $$\tilde{R}_{j} = \sum\limits_{j = k + 1}^{m} {\tilde{x}_{ij} }$$

Step 7: Evaluate the minimum value of $\tilde{R}_{j}$ and $\tilde{R}_{\min }$ by using the Eq. 15.

15 $$\tilde{R}_{\min } = \min_{j} \tilde{R}_{j} ;j = 1,2, \ldots ,n$$

Step 8: Obtain the relative significance of each alternative by using the Eq. 16.

16 $$\tilde{Q}_{j} = \tilde{P}_{j} + \frac{{\tilde{R}_{\min } \sum\nolimits_{j = 1}^{n} {\tilde{R}_{j} } }}{{\tilde{R}_{j} \sum\nolimits_{j = 1}^{n} {\frac{{\tilde{R}_{\min } }}{{\tilde{R}_{j} }}} }};j = 1,2,...,n$$

Step 9: Determine the value of optimality criteria $K$ by using the Eq. 17.

17 $$K = \max_{j} \tilde{Q}_{j} ;j = 1,2,...,n$$

Step 10: Determine the priority. Greater value of ${Q}_{j}$ for the alternative $j$ indicates a higher priority of the alternatives.

Step 11: Estimate the utility degree of the alternatives by using the Eq. 18.

18 $$N_{j} = \frac{{Q{}_{j}}}{{Q_{\max } }} \times 100;j = 1,2,...,n$$

### Application of the proposed TFWBMAO based fuzzy CRITIC-COPRAS approach

**Fig. 16:**
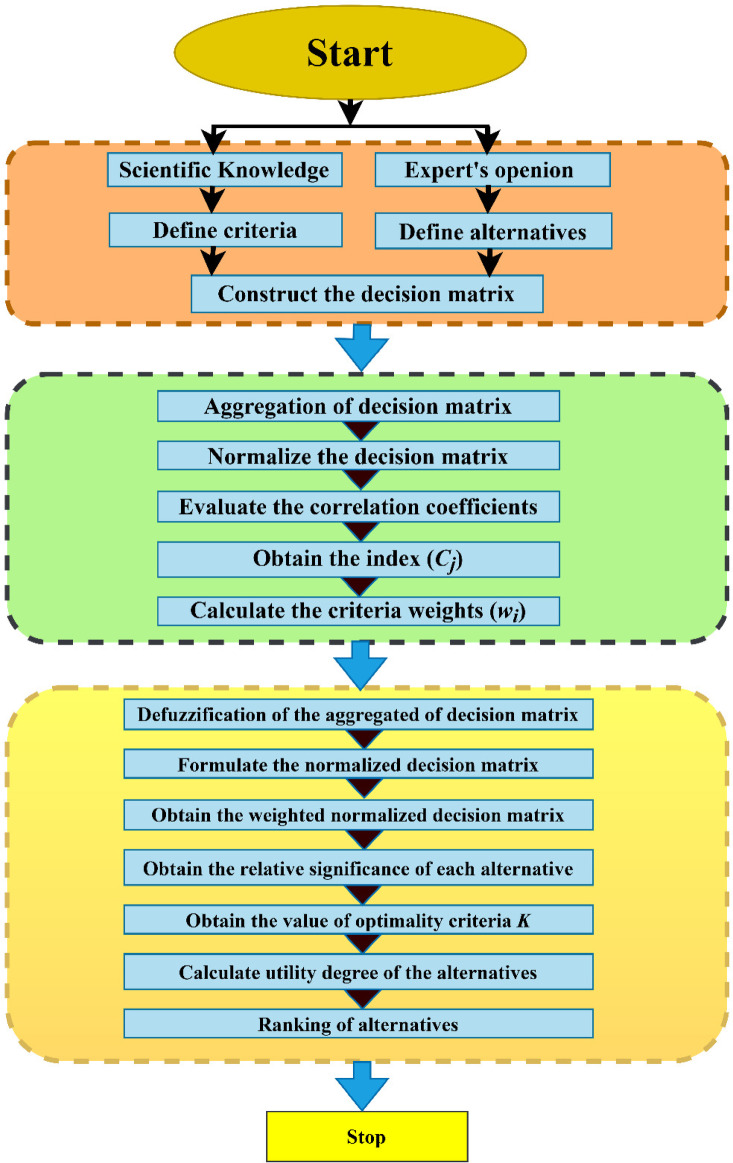
Flow chart of the proposed TFWBMAO based Fuzzy CRITIC-COPRAS approach.

**Fig. 17:**
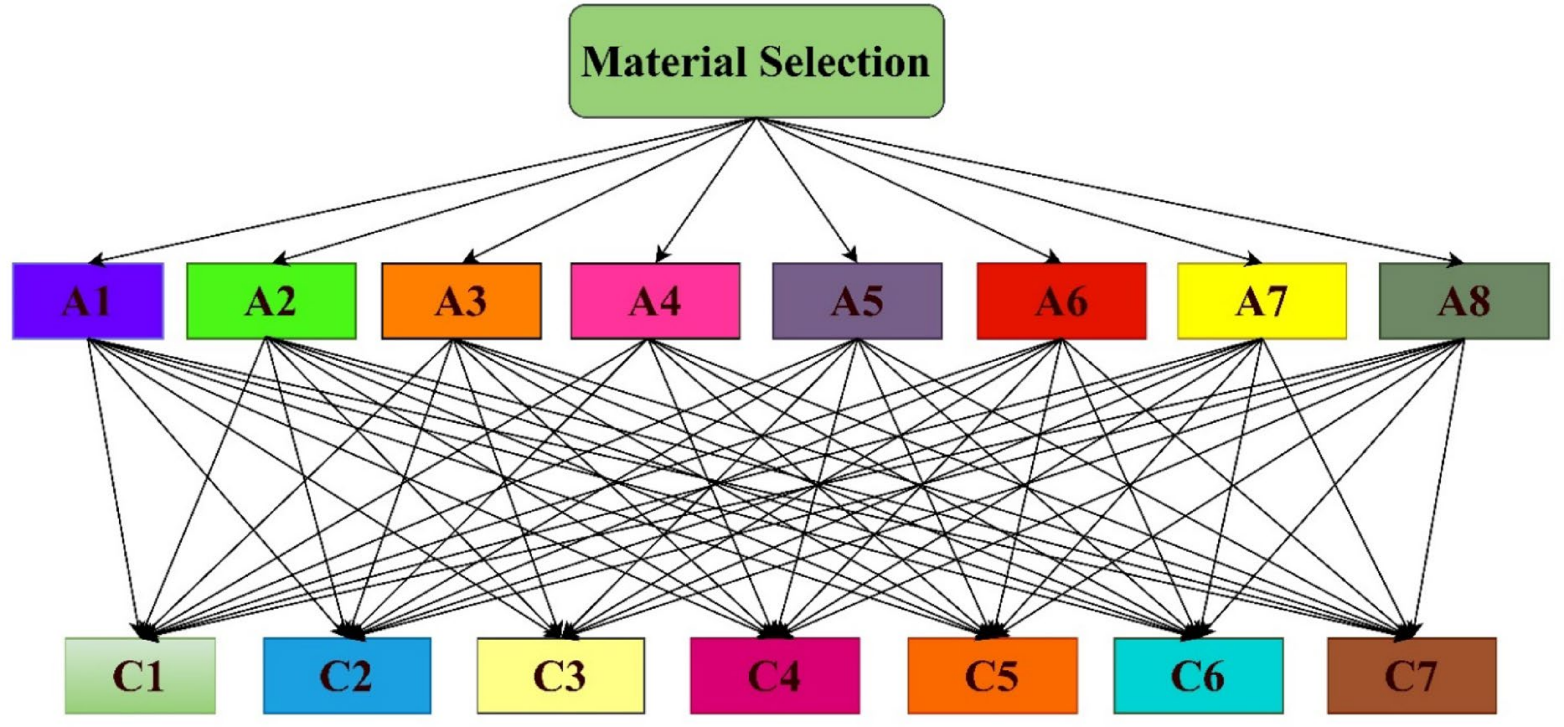
Problem order for the material selection.

In this section, the proposed TFWBMAO-based integrated CRITIC-COPRAS method has been applied for the ranking of the developed samples for structural applications. The flowchart for the proposed methodology is given in Fig. 16. The linguistic assessments were collected from the five (05) decision-makers $DMs$ namely ${DM}_{1}$,${DM}_{2}$,…….${DM}_{5}$ who are expertise in the field of composite and their applications in the form of a questionnaire for identifying a proper composition of biocomposites for structural applications among a set of eight (08) different alternatives and five (05) different criteria. Table 3 represent the details of the criteria for this study. In this connection, Fig. 17 provides the decision order of the proposed material selection problem. An equal weight has been assigned to the $DMs$ by considering their expertise and experience in the area of composites and their applications. The assessments given by the decision makers ($DMs$) for the structural application are furnished in Table 4 in the form of a triangular fuzzy scale of relative significance. The assessment provided by the ($DMs$) in linguistic terms of the provided in Table 5 in the form of $TFNs$. The linguistic valuation provided by the *DMs* to evaluate the developed samples of biocomposite is given in Table 6.

**Table 3:** Details of the criteria.

Criteria	Property	Details	Attributes
C1	Density	Influences the weight of the structural materials and stability	Beneficial
C2	Hardness	Determine the long-term durability and structural integrity	Beneficial
C3	Compressive strength	Determine the pressure required to break under compressive load	Beneficial
C4	Flexural strength	Determine the capacity to withstand external bending load	Beneficial
C5	Tensile strength	Determine the maximum stress against pulling or stretching	Beneficial
C6	Impact strength	Determine the capacity to resist sudden loading	Beneficial
C7	Sliding wear	Loss of materials during mechanical contact	Non-Beneficial

**Table 4:** Triangular fuzzy number based linguistic term.

Linguistic assessment	Linguistic value	Triangular fuzzy set
Very low	VL	(2,3,4)
Low	L	(3,4,5)
Moderate	M	(6,7,8)
High	H	(7,8,9)
Very high	VH	(8,9,9)

**Table 5:** Linguistic valuation of the DMs for alternatives.

		A 1	A 2	A 3	A 4	A 5	A 6	A 7	A 8
DM1	C1	(7,8,9)	(7,8,9)	(7,8,9)	(8,9,9)	(8,9,9)	(8,9,9)	(8,9,9)	(8,9,9)
	C2	(3,4,5)	(3,4,5)	(3,4,5)	(3,4,5)	(2,3,4)	(2,3,4)	(2,3,4)	(2,3,4)
	C3	(8,9,9)	(8,9,9)	(8,9,9)	(8,9,9)	(8,9,9)	(7,8,9)	(7,8,9)	(8,9,9)
	C4	(8,9,9)	(8,9,9)	(8,9,9)	(8,9,9)	(8,9,9)	(7,8,9)	(7,8,9)	(7,8,9)
	C5	(6,7,8)	(6,7,8)	(6,7,8)	(6,7,8)	(6,7,8)	(6,7,8)	(6,7,8)	(3,4,5)
	C6	(6,7,8)	(3,4,5)	(6,7,8)	(6,7,8)	(3,4,5)	(3,4,5)	(6,7,8)	(6,7,8)
	C7	(3,4,5)	(2,3,4)	(2,3,4)	(3,4,5)	(3,4,5)	(2,3,4)	(2,3,4)	(3,4,5)
DM2	C1	(7,8,9)	(7,8,9)	(7,8,9)	(8,9,9)	(8,9,9)	(8,9,9)	(8,9,9)	(8,9,9)
	C2	(3,4,5)	(3,4,5)	(3,4,5)	(3,4,5)	(2,3,4)	(2,3,4)	(2,3,4)	(2,3,4)
	C3	(8,9,9)	(8,9,9)	(8,9,9)	(8,9,9)	(8,9,9)	(7,8,9)	(7,8,9)	(8,9,9)
	C4	(8,9,9)	(8,9,9)	(8,9,9)	(8,9,9)	(8,9,9)	(7,8,9)	(7,8,9)	(7,8,9)
	C5	(6,7,8)	(6,7,8)	(6,7,8)	(6,7,8)	(6,7,8)	(3,4,5)	(6,7,8)	(6,7,8)
	C6	(6,7,8)	(6,7,8)	(3,4,5)	(6,7,8)	(6,7,8)	(3,4,5)	(6,7,8)	(6,7,8)
	C7	(3,4,5)	(2,3,4)	(2,3,4)	(3,4,5)	(3,4,5)	(2,3,4)	(2,3,4)	(3,4,5)
DM3	C1	(7,8,9)	(7,8,9)	(7,8,9)	(8,9,9)	(8,9,9)	(8,9,9)	(8,9,9)	(8,9,9)
	C2	(3,4,5)	(3,4,5)	(3,4,5)	(3,4,5)	(2,3,4)	(2,3,4)	(2,3,4)	(2,3,4)
	C3	(8,9,9)	(8,9,9)	(8,9,9)	(8,9,9)	(8,9,9)	(7,8,9)	(7,8,9)	(8,9,9)
	C4	(7,8,9)	(7,8,9)	(7,8,9)	(8,9,9)	(7,8,9)	(7,8,9)	(7,8,9)	(8,9,9)
	C5	(6,7,8)	(6,7,8)	(6,7,8)	(6,7,8)	(6,7,8)	(3,4,5)	(6,7,8)	(6,7,8)
	C6	(6,7,8)	(3,4,5)	(3,4,5)	(6,7,8)	(3,4,5)	(3,4,5)	(6,7,8)	(6,7,8)
	C7	(3,4,5)	(2,3,4)	(2,3,4)	(3,4,5)	(3,4,5)	(2,3,4)	(2,3,4)	(3,4,5)
DM4	C1	(7,8,9)	(7,8,9)	(7,8,9)	(8,9,9)	(8,9,9)	(8,9,9)	(8,9,9)	(8,9,9)
	C2	(2,3,4)	(2,3,4)	(2,3,4)	(2,3,4)	(3,4,5)	(3,4,5)	(3,4,5)	(3,4,5)
	C3	(8,9,9)	(8,9,9)	(8,9,9)	(8,9,9)	(8,9,9)	(7,8,9)	(7,8,9)	(8,9,9)
	C4	(8,9,9)	(8,9,9)	(8,9,9)	(8,9,9)	(8,9,9)	(7,8,9)	(7,8,9)	(7,8,9)
	C5	(6,7,8)	(6,7,8)	(3,4,5)	(6,7,8)	(3,4,5)	(6,7,8)	(6,7,8)	(6,7,8)
	C6	(6,7,8)	(3,4,5)	(3,4,5)	(6,7,8)	(3,4,5)	(3,4,5)	(6,7,8)	(6,7,8)
	C7	(2,3,4)	(3,4,5)	(2,3,4)	(3,4,5)	(3,4,5)	(3,4,5)	(2,3,4)	(2,3,4)
DM5	C1	(7,8,9)	(8,9,9)	(7,8,9)	(8,9,9)	(8,9,9)	(8,9,9)	(8,9,9)	(7,8,9)
	C2	(3,4,5)	(3,4,5)	(2,3,4)	(3,4,5)	(2,3,4)	(3,4,5)	(2,3,4)	(2,3,4)
	C3	(8,9,9)	(8,9,9)	(8,9,9)	(8,9,9)	(8,9,9)	(7,8,9)	(7,8,9)	(8,9,9)
	C4	(8,9,9)	(8,9,9)	(8,9,9)	(8,9,9)	(8,9,9)	(7,8,9)	(7,8,9)	(7,8,9)
	C5	(6,7,8)	(6,7,8)	(3,4,5)	(6,7,8)	(6,7,8)	(6,7,8)	(3,4,5)	(6,7,8)
	C6	(6,7,8)	(3,4,5)	(3,4,5)	(6,7,8)	(6,7,8)	(3,4,5)	(6,7,8)	(6,7,8)
	C7	(2,3,4)	(3,4,5)	(2,3,4)	(3,4,5)	(2,3,4)	(3,4,5)	(2,3,4)	(2,3,4)

**Table 6:** Assessment of the DMs for the criteria.

DM	C 1	C 2	C 3	C 4	C 5	C 6	C 7
DM1	(3,4,5)	(3,4,5)	(8,9,9)	(7,8,9)	(6,7,8)	(3,4,5)	(3,4,5)
DM2	(6,7,8)	(2,3,4)	(7,8,9)	(8,9,9)	(3,4,5)	(6,7,8)	(2,3,4)
DM3	(3,4,5)	(3,4,5)	(8,9,9)	(7,8,9)	(6,7,8)	(3,4,5)	(3,4,5)
DM4	(7,8,9)	(2,3,4)	(7,8,9)	(8,9,9)	(3,4,5)	(6,7,8)	(2,3,4)
DM5	(6,7,8)	(3,4,5)	(8,9,9)	(6,7,8)	(6,7,8)	(3,4,5)	(3,4,5)

The algorithm of the proposed TFWBM operator based CRITIC-COPRAS for ranking of the developed biocomposites for structural applications are as follows:

Step 1: Calculate the aggregated decision matrix by using TFWBMO and the results are given in Table 7.

**Table 7:** Aggregated decision matrix.

	C1	C2	C3	C4	C5	C6	C7
A1	(0.885438, 1.011929, 1.13842)	(0.35327, 0.48, 0.632456)	(1.011929, 1.13842, 1.13842)	(0.986306, 1.112834, 1.13842)	(0.758947, 0.885438, 1.011929)	(0.758947, 0.885438, 1.011929)	(0.327414, 0.454313, 0.581034)
A2	(0.910385, 1.036919, 1.13842)	(0.35327, 0.48, 0.60663)	(1.011929, 1.13842, 1.13842)	(0.986306, 1.112834, 1.13842)	(0.758947, 0.885438, 1.011929)	(0.448999, 0.576888, 0.704273)	(0.301993, 0.428952, 0.555698)
A3	(0.885438, 1.011929, 1.13842)	(0.327414, 0.454313, 0.581034)	(1.011929, 1.13842, 1.13842)	(0.986306, 1.112834, 1.13842)	(0.6, 0.727736, 0.855102)	(0.448999, 0.576888, 0.704273)	(0.252982, 0.379473, 0.505964)
A4	(1.011929, 0.974885, 1.13842)	(0.35327, 0.48, 0.60663)	(1.011929, 1.13842, 1.13842)	(1.011929, 1.13842, 1.13842)	(0.758947, 0.885438, 1.011929)	(0.758947, 0.885438, 1.011929)	(0.448999, 0.505964, 0.632456)
A5	(1.011929, 1.13842, 1.032279)	(0.277128, 0.40398, 0.53066)	(1.011929, 1.13842, 1.13842)	(0.986306, 1.112834, 1.13842)	(0.678823, 0.805978, 0.957497)	(0.523068, 0.651153, 0.778717)	(0.35327, 0.48, 0.60663)
A6	(1.011929, 1.13842, 1.13842)	(0.301993, 0.428952, 0.555698)	(0.885438, 1.011929, 1.13842)	(0.885438, 1.011929, 1.13842)	(0.6, 0.727736, 0.855102)	(0.379473, 0.505964, 0.632456)	(0.301993, 0.428952, 0.555698)
A7	(1.011929, 1.13842, 1.13842)	(0.277128, 0.40398, 0.53066)	(0.885438, 1.011929, 1.13842)	(0.885438, 1.011929, 1.13842)	(0.678823, 0.805978, 0.932952)	(0.758947, 0.885438, 1.011929)	(0.252982, 0.379473, 0.505964)
A8	(0.986306, 1.112834, 1.13842)	(0.277128, 0.40398, 0.53066)	(1.011929, 1.13842, 1.13842)	(0.910385, 1.036919, 1.13842)	(0.678823, 0.805978, 0.932952)	(0.758947, 0.885438, 1.011929)	(0.327414, 0.454313, 0.581034)

Step 2: Obtain the normalize decision matrix for the assessment of criteria weights by normalizing the beneficial and non-beneficial by using the Eqs. (2) and (3), respectively as given in Table 8.

**Table 8:** Normalized Decision matrix for criteria weights.

	C1	C2	C3	C4	C5	C6	C7
A1	0	1	1	0.797586	1	1	0.499649
A2	0.821645	0.898313	1	0.797586	1	0.186458	0.33012
A3	0.777779	0.594564	1	0.797586	0	0.186458	0
A4	0.856351	0.898313	1	1	1		1
A5	0.906765	0	0	0.797586	0.547987	0.382147	0.671451
A6	1	0.294831	0	0	0	0	0.33012
A7	1	0	0	0	0.496151	1	0
A8	0.955019	0	0	0.197398	0.496151	1	0.499649

Step 3: Calculate the correlation coefficients ${\rho }_{jk}$ between the criteria by using the Eq. 4 and the results are furnished in Table 9.

**Table 9:** Correlations coefficients between the attributes.

	C1	C2	C3	C4	C5	C6	C7
C1	1	− 0.63708	− 0.39427	− 0.44572	− 0.4587	− 0.29117	− 0.11266
C2	− 0.63708	1	0.439403	0.684637	0.548805	− 0.01037	0.249784
C3	− 0.39427	0.439403	1	0.825556	0.473111	0.130764	0.464332
C4	− 0.44572	0.684637	0.825556	1	0.534978	− 0.00844	0.48916
C5	− 0.4587	0.548805	0.473111	0.534978	1	0.530527	0.569649
C6	− 0.29117	− 0.01037	0.130764	− 0.00844	0.530527	1	0.324036
C7	− 0.11266	0.249784	0.464332	0.48916	0.569649	0.324036	1

Step 4: Calculate the standard deviation ${\sigma }_{j}$ of each attribute from the normalized decision matrix by using the Eq. 6 and the results are given in Table 10.

**Table 10:** Standard deviations of the normalized attributes.

Attributes	C1	C2	C3	C4	C5	C6	C7
${\sigma }_{j}$	0.329224	0.440133	0.46291	0.410053	0.416764	0.445492	0.334059

Step 5: Obtain the index (${C}_{j}$) by using Eq. 7.

Step 6: Calculate the weights of attributes ${w}_{j}$ by using the Eq. 8 and the criteria weight are given in Table 11.

**Table 11:** Weights of the attributes.

Attributes	C1	C2	C3	C4	C5	C6	C7
${c}_{j}$	2.745595	2.079549	1.879926	1.607337	1.584383	2.817581	1.341479
${w}_{j}$	0.195	0.148	0.133	0.114	0.113	0.200	0.095
Rank of the attributes	2	3	4	5	6	1	7

Step 7: Obtained the defuzzified aggregated matrix to crisp numbers as given in Table 12.

**Table 12:** Defuzzified aggregated decision matrix.

	C1	C2	C3	C4	C5	C6	C7
A1	0.716783	0.488575	1.096256	1.079187	0.885438	0.885438	0.454254
A2	1.028575	0.479967	1.096256	1.079187	0.885438	0.57672	0.428881
A3	1.011929	0.454254	1.096256	1.079187	0.727613	0.57672	0.379473
A4	1.041745	0.479967	1.096256	1.096256	0.885438	0.885438	0.52914
A5	1.060876	0.403923	1.096256	1.079187	0.814099	0.650979	0.479967
A6	1.096256	0.428881	1.011929	1.011929	0.727613	0.505964	0.428881
A7	1.096256	0.403923	1.011929	1.011929	0.805918	0.885438	0.379473
A8	1.079187	0.403923	1.096256	1.028575	0.805918	0.885438	0.454254

Step 8: Obtained the normalized decision matrix from defuzzified aggregated matrix by using the Eq. 11 as given in Table 13.

**Table 13:** Normalized decision matrix.

	C 1	C 2	C 3	C 4	C 5	C 6	C 7
A 1	0.088148	0.137883	0.127451	0.127482	0.13544	0.151302	0.128526
A 2	0.126491	0.135453	0.127451	0.127482	0.13544	0.098549	0.121347
A 3	0.124444	0.128197	0.127451	0.127482	0.111299	0.098549	0.107368
A 4	0.128111	0.135453	0.127451	0.129498	0.13544	0.151302	0.149715
A 5	0.130463	0.113993	0.127451	0.127482	0.124528	0.111238	0.135802
A 6	0.134814	0.121036	0.117647	0.119537	0.111299	0.086458	0.121347
A 7	0.134814	0.113993	0.117647	0.119537	0.123277	0.151302	0.107368
A 8	0.132715	0.113993	0.127451	0.121503	0.123277	0.151302	0.128526

Step 9: Obtained the weighted normalized decision matrix by using the Eq. 12 as given in Table 14.

**Table 14:** Weighted normalized decision matrix.

	C 1	C 2	C 3	C 4	C 5	C 6	C 7
A 1	0.017218	0.0204	0.017046	0.014578	0.015267	0.030329	0.012266
A 2	0.024708	0.02004	0.017046	0.014578	0.015267	0.019755	0.011581
A 3	0.024308	0.018967	0.017046	0.014578	0.012546	0.019755	0.010247
A 4	0.025024	0.02004	0.017046	0.014809	0.015267	0.030329	0.014289
A 5	0.025484	0.016865	0.017046	0.014578	0.014037	0.022298	0.012961
A 6	0.026334	0.017907	0.015735	0.013669	0.012546	0.017331	0.011581
A 7	0.026334	0.016865	0.015735	0.013669	0.013896	0.030329	0.010247
A 8	0.025924	0.016865	0.017046	0.013894	0.013896	0.030329	0.012266

Step 10: Using the Eq. 16 by using the relative significance of the alternatives.

Step. 11: Calculate the utility degree of the alternatives by using the Eq. 18 to obtain the rankings of the alternatives as given in Table 15.

**Table 15:** Ranking of the alternatives.

Alternative	A1	A2	A3	A4	A5	A6	A7	A8
Utility degree ${N}_{j}$	0.954268	0.933376	0.91364	1	0.915402	0.873905	0.986387	0.977811
Ranking	4	5	7	1	6	8	2	3

## Results and discussion

The work has successfully demonstrated the development of an integrated fuzzy CRITIC-COPRAS for the material selection of biocomposites. The result of the fuzzy CRITIC method has observed the ranking sequence of criteria as C6 > C1 > C2 > C3 > C4 > C5 > C7. It denotes that the impact is the most significant factor and density is the second most significant factor for structural applications of the biocomposites whereas the sliding wear is the least significant factor.

**Fig. 18:**
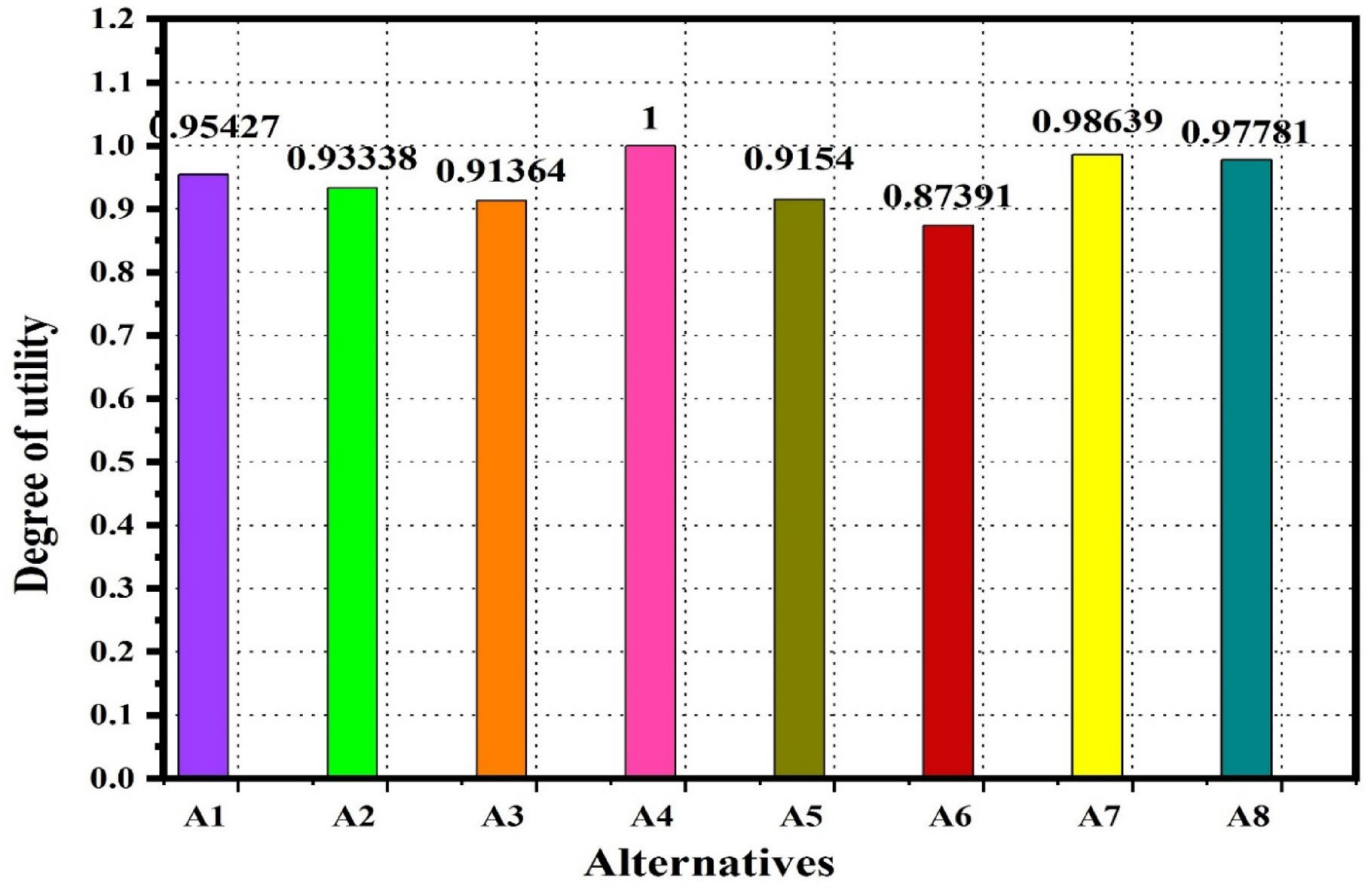
Ranking of alternatives.

**Fig. 19:**
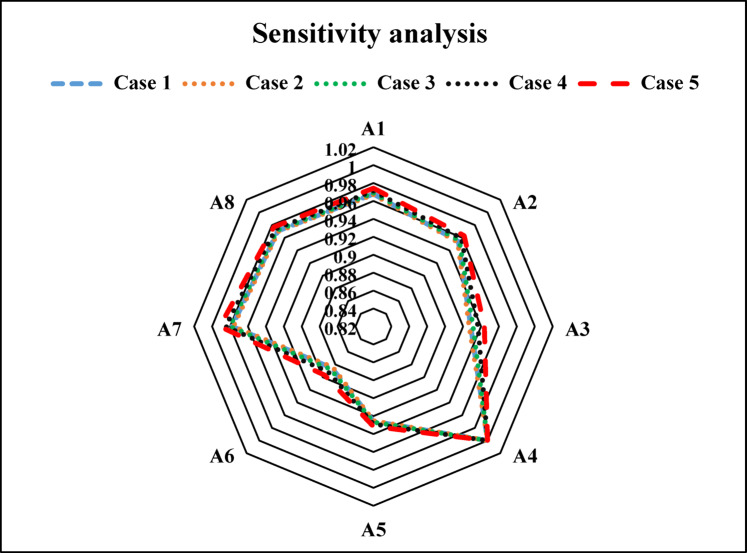
Plot for the sensitivity analysis for the cases of criteria weight.

The structural components are subjected to the sudden impact loadings and vibrations during service. A good impact strength ensures the capacity of the composite to absorb and dissipate energy without failure and is important to prevent the failure of the materials due to the dynamic forces. The high impact strength maintains the load-bearing capacity of the components and improves the overall lifespan and durability of the material. Therefore, this property dominates for structural applications over other mechanical parameters. The density of the composites influences the mechanical therefore the suitability of the material. It affects the specific strength i.e. strength-to-weight ratio. It is important in applications where the concern for load-bearing is of prime importance. The structural components are intended to carry loads and resist deformation and there is an almost negligible relative surface motion making the sliding wear a least important property for structural applications. The results of the integrated fuzzy CRITIC-COPRAS method plotted in Fig. 18 have demonstrated the ranking of the alternatives follows the sequences as A4 > A7 > A8 > A1 > A2 > A5 > A3 > A6. The biocomposite B20P80 is the most suitable candidate for structural applications having a utility degree of 1. The reinforcement of 20 wt.% of BPP with 80 wt.% of PLA provides good strength and utilizes the optimal amount of agro waste-derived biofillers therefore, is preferable from a techno-environmental point of view. The work is correlated with work where MCDM was employed to identify a phase change materials for energy application selections ^114^. The identified optimal composites can be easily processed by any conventional methods which favour there production of industrial scale. The biofillers serves as an eco-friendly and effective reinforcement which improves the interfacial bonding with PLA without compromising the processability. The utilizations of biofillers promotes circular economy models reduces the cost of materials and impact on environment. The optimal composites possess a good mechanical strength and wear properties therefore are durable. The alternative A7 i.e. biocomposite P85BO15 is the second most preferable material for structural applications as the incorporation of the 15 wt.% of the hybrid biofillers with 85 wt.% of the PLA slightly reduces the performance of the composite due to less fraction of the composites. It is observed that the best alternative gives an impact strength of 20,000 kJ/m^2^ and a density of 0.32 g/cm^3^ which are the first and second most important considerations for structural application of the composite. A similar observation of the alternative A7 has found an impact strength of 21,000 kJ/m^2^ and density of 0.41 g/cm^3^ which is slightly higher than the alternative A4 however the significant reduction in the mechanical strength such as compressive strength, flexural strength, and tensile strength from 68.81 to 43.92 MPa, 47.20 to 36.8 MPa and 32.80 to 12.9 MPa, respectively reduces the priority index of the biocomposite P85BO15. The identified best composite utilizes the optimal fraction of biofillers which is derived from waste without compromising the workability for the structural applications as per the obtained criteria weight. Moreover, the higher impact factor is a crucial factor as the impact strength has identified as the most important while the density has identifies as the third important property for the workability the composites for structural applications. This work can be compared with the work where the Evaluation based on distance from Average Solution (EDAS) method was employed for the selections of best composites from a given set of materials developed by the reinforcement of date palm fiber reinforced containing powdered activated carbon in concrete for building constructions ^115^ The resulted properties of the biocomposites has verifed the feasibility of their applications in structural applications. Overall, the work has provided a reliable and efficient approach to material selection. Moreover, the utilization of fruit waste-derived biofillers and biodegradable polymers in biocomposites is a sustainable alternative to conventional materials. The sensitive analysis has been provided for the different case of the criteria weight as given in Table 16 and the rankings for the different cases has been furnished in Table 17 and plotted as Fig. 19 shows that ranking results is unaffected by the change in criteria weights for the considered cases therefore verifies the robustness of the material ranking against variations in criteria weights.

**Table 16:** Cases for the criteria weight.

Case	Details
Case 1	Equal weight to each criteria
Case 2	90% weight to beneficial criteria and 10% weight to non-beneficial criteria
Case 3	80% weight to beneficial criteria and 20% weight to non-beneficial criteria
Case 4	70% weight to beneficial criteria and 30% weight to non-beneficial criteria
Case 5	60% weight to beneficial criteria and 40% weight to non-beneficial criteria

**Table 17:** Rankings for the cases of the criteria weight.

Case	Rankings
Case 1	A4 > A7 > A8 > A1 > A2 > A3 > A5 > A6
Case 2	A4 > A7 > A8 > A1 > A2 > A3 > A5 > A6
Case 3	A4 > A7 > A8 > A1 > A2 > A3 > A5 > A6
Case 4	A4 > A7 > A8 > A1 > A2 > A3 > A5 > A6
Case 5	A4 > A7 > A8 > A1 > A2 > A3 > A5 > A6

## Conclusion and future scope

The materials selection is a crucial step in the design and manufacturing of any product. The proper selection of material increases the workability of the product, reduces the failures, and increases the overall life cycle of the product. The implementation of fuzzy decision-making techniques in material selection is gaining significance. The work proposed an integrated fuzzy CRITIC- COPRAS approach for material selection of biocomposites. The biocomposites are developed by using a biopolymer namely, PLA with the reinforcement of hybrid biofillers of orange and banana peel powder. The proposed framework assisted in the integration of the different opinions of the decision-makers through the aggregation of the opinions by using suitable aggregation operators and effectively handles the hesitancy or vagueness in the decision-making. It is observed that biofillers can be an effective reinforcement in polymers to increase the workability of the composites. However, the behaviour of the material for the different properties varies irregularly with the compositions. The developed composites can effectively replace the conventional structural materials by an alternative and innovative material. CRITIC approach has identified that impact strength is the most significant factor for the materials subjected to the structural applications whereas the specific wear is less preferable criteria. The integrated mathematical approach has effective rank the alternative and has identified the incorporation of 20 wt. % of BPP in 80 wt. % of PLA as the most suitable compositions for structural applications.

The proposed integrated can effectively rank the alternatives under a given set of criteria. The developed mathematical model can be applied for material section in the design and development of composites. The proposed methodology can effectively rank the alternative in situation where the conditions of hesitancy exist. The research provided the community with a novel and alternative composite for structural applications along with a systematic and logical approach for materials selection to assist in the design and development of materials of such composite on commercial scale. The work is helpful for the researcher and authorities engaged in the domain of sustainability. The work favours for the large-scale production and commercialization of sustainable composites by providing an effective and reliable technique for material selection. The work is pivotal from different points of view including techno, eco, and environmental. The efficacy of the approach makes it a suitable for a broader array of materials selection and promotes sustainability by using fruit-derived biofillers and biodegradable materials to develop green and sustainable materials for structural applications.

In the present work, only a limited number of composite samples are developed by utilizing a biopolymer (polylactic acid) and orange and banana peel powder. Instead, there are a number of fruits and agro-waste-derived biofillers whose potential is still not yet explored. Therefore, future work is suggested to develop biocomposites by using different types of matrices and reinforcement with a better mixture proportions. It is anticipated that the incorporations of a suitable reinforcement in matrix can improve the performance of biocomposites up to 30% to 40%. Moreover, here only seven (07) criteria is considered whereas there are a several other properties which requires investigations to explore the workability of such materials. In the context of materials selections instead of triangular fuzzy numbers, spherical fuzzy sets can be implemented for the same set of methodology. Also, an integrated objective-subjective criteria-weighted method and other uncertainty theories can be implemented.

## Data Availability

Data is provided within the manuscript.

## References

[1] Facts, P. An analysis of European plastics production, demand and waste data. *Plast Europe* (2019).

[2] Borrelle, S. B. et al. Predicted growth in plastic waste exceeds efforts to mitigate plastic pollution. *Science* **369**, 1515–1518. 10.1126/science.aba3656 (2020). 32943526 10.1126/science.aba3656

[3] Dube, E. & Okuthe, G. E. Plastics and micro/nano-plastics (MNPs) in the environment: Occurrence, impact, and toxicity. *Int. J. Environ. Res. Public Health* **20**, 6667. 10.3390/ijerph20176667 (2023). 37681807 10.3390/ijerph20176667 PMC10488176

[4] Lin, J. et al. Effect of degradable microplastics, biochar and their coexistence on soil organic matter decomposition: A critical review. *TrAC Trends Anal. Chem.* **183**, 118082. 10.1016/j.trac.2024.118082 (2025).

[5] Felix Sahayaraj, A., Muthukrishnan, M. & Ramesh, M. Experimental investigation on physical, mechanical, and thermal properties of jute and hemp fibers reinforced hybrid polylactic acid composites. *Polym. Compos.* **43**, 2854–2863. 10.1002/pc.26581 (2022).

[6] Hariprasad, P. *et al.* In *International conference on eco-friendly fibers and polymeric materials.* 281–290 (Springer).

[7] Ramkumar, C. *et al.* In *International conference on eco-friendly fibers and polymeric materials.* 569–583 (Springer).

[8] Ramesh, M., Sahayaraj, A. F. & Selvan, M. T. In *Natural fiber-reinforced PLA composites* 3–23 (Elsevier, 2025). 10.1016/B978-0-323-95247-7.00008-8.

[9] Chougala, V., Gowda, A. C., Nagaraja, S. & Ammarullah, M. I. Effect of chemical treatments on mechanical properties of sugarcane bagasse (Gramineae *Saccharum officinarum* L.) fiber based biocomposites: A review. *J. Nat. Fibers* **22**, 2445571. 10.1080/15440478.2024.2445571 (2025).

[10] Keerthiveettil Ramakrishnan, S., Vijayananth, K., Arivendan, A. & Ammarullah, M. I. Influence of Artocarpus hirsutus (AH) cellulose micro fiber, bamboo fiber in thermoplastic biocomposites. *Sci. Rep.* **15**, 4611. 10.1038/s41598-025-88058-5 (2025). 39920190 10.1038/s41598-025-88058-5 PMC11805915

[11] Ahmad, A. et al. Development and characterisation of eco-friendly hybrid polymer composites from palm oil empty fruit bunch (EFB) fibre and glass fiber reinforced polyester for biomedical applications. *Mater. Technol.* **40**, 2512017. 10.1080/10667857.2025.2512017 (2025).

[12] Roseno, S. et al. The effects of carbon fiber surface treatment by oxidation process for enhanced mechanical properties of carbon fiber/epoxy composites for biomedical application. *AIP Adv.*10.1063/5.0183153 (2024).

[13] Basavaraju, B. et al. Influence of suspended cenospheres on the mechanical characteristics and wear loss of natural fiber-reinforced hybrid composites: Implications for biomedical applications and sustainable material management. *RSC Adv.* **14**, 33332–33344. 10.1039/D4RA06223J (2024). 39439842 10.1039/d4ra06223j PMC11494284

[14] Anand, P. B. et al. Synthesis and characterization of mechanical properties of dammar gum-epoxy bio-composites with areca nut husk and banana fiber as reinforcements for biomedical applications. *J. Chem. Eng. Jpn.* **57**, 2438111. 10.1080/00219592.2024.2438111 (2024).

[15] Ammarullah, M. I. et al. A review of enhanced total hip prosthesis design and material bearing combination to accommodate Muslim prayer (Salat) movements: Biomechanical, biotribological, and biological perspectives. *Tribol. Int.*10.1016/j.triboint.2025.110518 (2025).

[16] Ammarullah, M. I., Hidayat, T., Lamura, M. D. P. & Jamari, J. Relationship between deformation and running-in wear on hard-on-hard bearings from metal, ceramic, and diamond materials for total hip prosthesis. *J. Tribol.* **38**, 69–81 (2023).

[17] Ammarullah, M. I. et al. Tresca stress study of CoCrMo-on-CoCrMo bearings based on body mass index using 2D computational model. *J. Tribol.* **33**, 31–38 (2022).

[18] Sinha, A. K. S., Narang, H. K. & Bhattacharya, S. In *Materials Science Forum.* 291–295 (Trans Tech Publ). 10.4028/www.scientific.net/MSF.978.291 2020.

[19] Sinha, A. K. et al. A review on mechanical properties of natural fibre reinforced PLA composites. *Curr. Mater. Sci. Formerly Recent Patents Mater. Sci.* **16**, 365–375. 10.2174/2666145415666211228163914 (2023).

[20] Akbar, I., Basri, H., Yanis, M. & Ammarullah, M. I. Optimization of PLA/Mg/PEG biocomposite filaments for 3D-printed bone scaffolds using response surface methodology (RSM). *Adv. Manuf. Polym. Compos. Sci.* **11**, 2448648. 10.1080/20550340.2024.2448648 (2025).

[21] Vasu, V. K., Anand, P. B., Nagaraja, S. & Ammarullah, M. I. Mechanical and fracture property optimization of graphene-SiO_2_-reinforced epoxy-PLA nanocomposites for biomedical applications. *Results Chem.* **13**, 102040. 10.1016/j.rechem.2025.102040 (2025).

[22] Lekrine, A. et al. Fiber treatment impact on the thermal behavior of biomass/palm-fibers polylactic-acid hybrid biocomposites. *Mater. Chem. Phys.* **338**, 130651. 10.1016/j.matchemphys.2025.130651 (2025).

[23] Dembri, I. et al. Structural and thermal properties of Alkali-treated biomass fibers and *W. robusta* waste reinforced PLA hybrid biocomposites. *Case Stud. Therm. Eng.*10.1016/j.csite.2025.106170 (2025).

[24] Dembri, I. et al. Effect of alkaline treatment on the thermo-physicochemical and mechanical properties of biochar powder/Washingtonia robusta fibers/PLA hybrid biocomposites. *J. Market. Res.* **33**, 9735–9751. 10.1016/j.jmrt.2024.12.018 (2024).

[25] Lekrine, A. et al. Thermomechanical and structural analysis of green hybrid composites based on polylactic acid/biochar/treated *W. filifera* palm fibers. *J. Mater. Res. Technol.* **30**, 9656–9667. 10.1016/j.jmrt.2024.06.033 (2024).

[26] Kurien, R. A. et al. Comparative mechanical properties of duck feather–jute fiber reinforced hybrid composites. *Trans. Indian Inst. Met.* **76**, 2575–2580. 10.1007/s12666-023-03015-y (2023).

[27] Kurien, R. A. *et al.* In *International conference on advances in materials processing & manufacturing applications.* 375–384 (Springer).

[28] Kurien, R. A. et al. A study on vetiver fiber and lemongrass fiber reinforced composites. *Mater. Today Proc.* **68**, 2640–2645. 10.1016/j.matpr.2022.09.563 (2022).

[29] Kurien, R. A. et al. Agave-jute fiber–reinforced hybrid composite for lightweight applications: Effect of hybridization. *Biomass Convers. Biorefinery* **15**, 12241–12254. 10.1007/s13399-024-05984-6 (2025).

[30] Kurien, R. A. et al. Comparative mechanical and morphological characteristics of an innovative hybrid composite of vetiver and jute. *J. Polym. Res.* **31**, 356. 10.1007/s10965-024-04208-9 (2024).

[31] Raja, R., Nagarjun, J., Kurien, R. A. & Jannet, S. 3D printing of tailored polymer composites: Enhancing sustainability with banana and pineapple fiber particle reinforcements. *Eng. Res. Express* **7**, 025442. 10.1088/2631-8695/addb2c (2025).

[32] Kurien, R. A., Selvaraj, D. P., Sekar, M. & Koshy, C. P. In *IOP conference series: materials science and engineering.* 012064 (IOP Publishing).10.1088/1757-899X/872/1/012064 2020.

[33] Kurien, R. A. et al. Chicken feather fiber reinforced composites for sustainable applications. *Mater. Today Proc.* **58**, 862–866. 10.1016/j.matpr.2021.10.400 (2022).

[34] Kurien, R. A., Anil, M. M., Mohan, S. S. & Thomas, J. A. Natural fiber composites as sustainable resources for emerging applications-a review. *Mater. Today Proc.*10.1016/j.matpr.2023.04.363 (2023).

[35] Kurien, R. A. et al. Fabrication, properties, and morphologies of novel acacia-jute hybrid polymer composites. *J. Compos. Sci.* **9**, 316. 10.3390/jcs9070316 (2025).

[36] Kurien, R. A., Selvaraj, D. P., Sekar, M. & Preno, C. Tribological and mechanical performance characteristics of Epoxy-Resin composites Reinforced with Multi-walled Carbon nanotubes for sustainable applications. *J. Green Eng.* **10**, 8859–8873 (2020).

[37] Chu, C.-S., Yusuf, A. A. & Ammarullah, M. I. High-performance ratiometric optical oxygen sensor fabricated via 3D-printed silicone for biomedical applications. *Talanta Open*10.1016/j.talo.2025.100513 (2025).

[38] Ammarullah, M. I. Integrating finite element analysis in total hip arthroplasty for childhood hip disorders: Enhancing precision and outcomes. *World J. Orthopedics* **16**, 98871. 10.5312/wjo.v16.i1.98871 (2025). 10.5312/wjo.v16.i1.98871 PMC11752482 39850035

[39] Safi’ai, A., Ammarullah, M. I., Baharuddin, M. H., Faidzul Hassan, N. S. & Ramlee, M. H. Evaluation of mechanical properties, color stability, and cleaning efficacy of biomed clear resin-based dental aligners. *Eng. Rep.* **7**, e70052. 10.1002/eng2.70052 (2025).

[40] Ismail, R., Fitriyana, D. F., Nugraha, F. W., Bayuseno, A. P. & Ammarullah, M. I. Investigation of the influence of 3D printing parameters on the properties of interference screws made of PLA/PCL/HA biocomposite filaments. *Mater. Technol.* **40**, 2443598. 10.1080/10667857.2024.2443598 (2025).

[41] James, D. J. D. et al. Chemically treated Acacia nilotica filler-reinforced epoxy composites: tribological studies and optimization of process parameters. *Chem. Pap.* **78**, 7395–7407. 10.1007/s11696-024-03601-4 (2024).

[42] Selvaraj, V. K. et al. Sustainable additive manufacturing of recycled Rigid PU/PLA composites via filament extrusion for enhanced mechanical and acoustic properties. *Results Eng.* **106**, 365. 10.1016/j.rineng.2025.106365 (2025).

[43] Vinod, A., Tengsuthiwat, J., Vijay, R., Sanjay, M. & Siengchin, S. Advancing additive manufacturing: 3D-printing of hybrid natural fiber sandwich (Nona/soy-PLA) composites through filament extrusion and its effect on thermomechanical properties. *Polym. Compos.* **45**, 7767–7789. 10.1002/pc.28302 (2024).

[44] Ayyappan, V., Rangappa, S. M., Tengsuthiwat, J., Fiore, V. & Siengchin, S. Investigation of thermo-mechanical and viscoelastic properties of 3D-printed Morinda citrifolia particle reinforced poly (lactic acid) composites. *Polym. Compos.* **45**, 5372–5385. 10.1002/pc.28133 (2024).

[45] Nagarjun, J., Kanchana, J., Rajeshkumar, G. & Anto Dilip, A. Enhanced mechanical characteristics of polylactic acid/tamarind kernel filler green composite filament for 3D printing. *Polym. Compos.* **44**, 7925–7940. 10.1002/pc.27676 (2023).

[46] Lage-Rivera, S., Ares-Pernas, A., Dopico-García, M. S., Covas, J. & Abad, M. J. Comparing lignin and spent coffee grounds as bio-fillers in PLA 3D-printable filaments. *Polym. Compos.* **45**, 14566–14579. 10.1002/pc.28782 (2024).

[47] Kringel, D. H., Dias, A. R. G., Zavareze, E. D. R. & Gandra, E. A. Fruit wastes as promising sources of starch: Extraction, properties, and applications. *Starch-Stärke* **72**, 1900200. 10.1002/star.201900200 (2020).

[48] Malav, L. C. et al. A review on municipal solid waste as a renewable source for waste-to-energy project in India: Current practices, challenges, and future opportunities. *J. Clean. Prod.* **277**, 123227. 10.1016/j.jclepro.2020.123227 (2020).

[49] Satari, B. & Karimi, K. Citrus processing wastes: Environmental impacts, recent advances, and future perspectives in total valorization. *Resour. Conserv. Recycl.* **129**, 153–167. 10.1016/j.resconrec.2017.10.032 (2018).

[50] Paggiola, G. et al. Can bio-based chemicals meet demand? Global and regional case-study around citrus waste-derived limonene as a solvent for cleaning applications. *Biofuels Bioprod. Biorefin.* **10**, 686–698. 10.1002/bbb.1677 (2016).

[51] Aboagye, D., Banadda, N., Kiggundu, N. & Kabenge, I. Assessment of orange peel waste availability in Ghana and potential bio-oil yield using fast pyrolysis. *Renew. Sustain. Energy Rev.* **70**, 814–821. 10.1016/j.rser.2016.11.262 (2017).

[52] Rathinavel, S. & Saravanakumar, S. Development and analysis of poly vinyl alcohol/orange peel powder biocomposite films. *J. Nat. Fibers* **18**, 2045–2054. 10.1080/15440478.2019.1711285 (2021).

[53] Pathak, P. D., Mandavgane, S. A. & Kulkarni, B. D. Fruit peel waste: Characterization and its potential uses. *Curr. Sci.* 444–454. https://www.jstor.org/stable/26294001 (2017).

[54] Platonovskiy, N. G., Ibrasheva, L. R., Obukhova, N. I., Puchkova, O. S. & Babkina, A. V. In *Sustainable development of the agrarian economy based on digital technologies and smart innovations* 25–30 (Springer). 10.1007/978-3-031-51272-8_5 (2024).

[55] El Barnossi, A., Moussaid, F. & Housseini, A. I. Tangerine, banana and pomegranate peels valorisation for sustainable environment: A review. *Biotechnol. Rep.* **29**, e00574. 10.1016/j.btre.2020.e00574 (2021). 10.1016/j.btre.2020.e00574 PMC7758358 33376681

[56] Yi, Z. et al. Changes in hemicellulose metabolism in banana peel during fruit development and ripening. *Plant Physiol. Biochem.* **215**, 109025. 10.1016/j.plaphy.2024.109025 (2024). 39142014 10.1016/j.plaphy.2024.109025

[57] Mohsin, A. et al. Advances in sustainable approaches utilizing orange peel waste to produce highly value-added bioproducts. *Crit. Rev. Biotechnol.* **42**, 1284–1303. 10.1080/07388551.2021.2002805 (2022). 34856847 10.1080/07388551.2021.2002805

[58] Sambudi, N. S., Lin, W. Y., Harun, N. Y. & Mutiari, D. Modification of poly (lactic acid) with orange peel powder as biodegradable composite. *Polymers* **14**, 4126. 10.3390/polym14194126 (2022). 36236074 10.3390/polym14194126 PMC9570532

[59] Saini, K., Matsagar, V. A. & Kodur, V. R. Recent advances in the use of natural fibers in civil engineering structures. *Constr. Build. Mater.* **411**, 134364. 10.1016/j.conbuildmat.2023.134364 (2024).

[60] Ramesh, M., Palanikumar, K. & Reddy, K. H. Plant fibre based bio-composites: Sustainable and renewable green materials. *Renew. Sustain. Energy Rev.* **79**, 558–584. 10.1016/j.rser.2017.05.094 (2017).

[61] Ahmad, W., McCormack, S. J. & Byrne, A. Biocomposites for sustainable construction: A review of material properties, applications, research gaps, and contribution to circular economy. *J. Build. Eng.*10.1016/j.jobe.2025.112525 (2025).

[62] Livne, A., Wösten, H. A., Pearlmutter, D. & Gal, E. Fungal mycelium bio-composite acts as a CO_2_-sink building material with low embodied energy. *ACS Sustain. Chem. Eng.* **10**, 12099–12106. 10.1021/acssuschemeng.2c01314 (2022).

[63] Olonisakin, K. et al. Key improvements in interfacial adhesion and dispersion of fibers/fillers in polymer matrix composites; focus on pla matrix composites. *Compos. Interfaces* **29**, 1071–1120. 10.1080/09276440.2021.1878441 (2022).

[64] Soni, A., Chakraborty, S., Das, P. K. & Saha, A. K. Materials selection of reinforced sustainable composites by recycling waste plastics and agro-waste: An integrated multi-criteria decision making approach. *Constr. Build. Mater.* **348**, 128608. 10.1016/j.conbuildmat.2022.128608 (2022).

[65] Mousavi-Nasab, S. H. & Sotoudeh-Anvari, A. A comprehensive MCDM-based approach using TOPSIS, COPRAS and DEA as an auxiliary tool for material selection problems. *Mater. Des.* **121**, 237–253. 10.1016/j.matdes.2017.02.041 (2017).

[66] Bhaskar, A. S. & Khan, A. Comparative analysis of hybrid MCDM methods in material selection for dental applications. *Expert Syst. Appl.* **209**, 118268. 10.1016/j.eswa.2022.118268 (2022).

[67] Hosouli, S. et al. A Multi-Criteria decision making (MCDM) methodology for high temperature thermochemical storage material selection using graph theory and matrix approach. *Mater. Des.* **227**, 111685. 10.1016/j.matdes.2023.111685 (2023).

[68] Zeng, D. et al. Material selection of titanium alloy pipelines considering multi-criteria in an acidic environment based on analytic hierarchy process. *Int. J. Pressure Vessels Piping*10.1016/j.ijpvp.2025.105461 (2025).

[69] Sahoo, D., Parida, P. K., Baral, S. P. & Pati, B. An innovative aggregation operator for enhanced decision-making: A study on interval-valued Pythagorean fuzzy soft sets in material selection. *Appl. Soft Comput.* **172**, 112888. 10.1016/j.asoc.2025.112888 (2025).

[70] Xie, G. et al. A hybrid multi-stage decision-making method with probabilistic interval-valued hesitant fuzzy set for 3D printed composite material selection. *Eng. Appl. Artif. Intell.* **123**, 106483. 10.1016/j.engappai.2023.106483 (2023).

[71] Yadav, R., Singh, M., Meena, A., Lee, S.-Y. & Park, S.-J. Selection and ranking of dental restorative composite materials using hybrid Entropy-VIKOR method: An application of MCDM technique. *J. Mech. Behav. Biomed. Mater.* **147**, 106103. 10.1016/j.jmbbm.2023.106103 (2023). 37690292 10.1016/j.jmbbm.2023.106103

[72] Haq, R. S. U., Saeed, M., Mateen, N., Siddiqui, F. & Ahmed, S. An interval-valued neutrosophic based MAIRCA method for sustainable material selection. *Eng. Appl. Artif. Intell.* **123**, 106177. 10.1016/j.engappai.2023.106177 (2023).

[73] Le Roux, D., Olivès, R. & Neveu, P. Combining entropy weight and TOPSIS method for selection of tank geometry and filler material of a packed-bed thermal energy storage system. *J. Clean. Prod.* **414**, 137588. 10.1016/j.jclepro.2023.137588 (2023).

[74] Akgün, H., Yapıcı, E., Özkan, A., Günkaya, Z. & Banar, M. A combined multi-criteria decision-making approach for the selection of carbon-based nanomaterials in phase change materials. *J. Energy Storage* **60**, 106619. 10.1016/j.est.2023.106619 (2023).

[75] Ajithkumar, A. & GaneshKumar, P. A systematic framework for the optimum selection of organic PCM in sustainable solar drying process: A multi-criteria decision-making methodology. *J. Energy Storage* **116**, 116080. 10.1016/j.est.2025.116080 (2025).

[76] Xie, G. et al. A behavior three-way decision approach under interval-valued triangular fuzzy numbers with application to the selection of additive manufacturing composites. *Eng. Appl. Artif. Intell.* **137**, 109214. 10.1016/j.engappai.2024.109214 (2024).

[77] Prabakar, P. et al. Production of MWCNTs from plastic wastes: Method selection through Multi-Criteria Decision-Making techniques. *J. Taiwan Inst. Chem. Eng.* **169**, 106000. 10.1016/j.jtice.2025.106000 (2025).

[78] Kumari, A. & Acherjee, B. Material selection for milling cutter inserts in high-speed machining applications using the CARCACS method. *Results Eng.*10.1016/j.rineng.2025.105063 (2025).

[79] Negi, A. et al. A hybrid CRITIC-MAIRCA framework for optimal phase change material selection in solar distillation systems. *Int. J. Thermofluids* **27**, 101167. 10.1016/j.ijft.2025.101167 (2025).

[80] Singh, T. et al. Selection of automotive brake friction composites reinforced by agro-waste and natural fiber: An integrated multi-criteria decision-making approach. *Results Eng.* **22**, 102030. 10.1016/j.rineng.2024.102030 (2024).

[81] Yiow, R. V., Mansor, M. R. & Shaharuzaman, M. A. Natural fibre composite selection for two-stroke marine engine under-piston door using hybrid AHP and TOPSIS methods. *Int. J. Lightweight Mater. Manuf.* **8**, 66–73. 10.1016/j.ijlmm.2024.07.006 (2025).

[82] Maidin, N., Sapuan, S. & Mastura, M. Materials selection of thermoplastic matrices of natural fibre composites for cyclist helmet using an integration of DMAIC approach in six sigma method together with grey relational analysis approach. *J. Renew. Mater.* **11**, 2381. 10.32604/jrm.2023.026549 (2023).

[83] Zakeri, S., Chatterjee, P., Cheikhrouhou, N., Konstantas, D. & Yang, Y. MUTRISS: A new method for material selection problems using MUltiple-TRIangles scenarios. *Expert Syst. Appl.* **228**, 120463. 10.1016/j.eswa.2023.120463 (2023).

[84] Cañete, R. C., Picardo, A., Trueba, P., Torres, Y. & Peralta, E. A new multi-criteria decision-making approach for the design and selection of materials and manufacturing processes of toys for children with autism. *Mater. Today Commun.*10.1016/j.mtcomm.2024.109709 (2024).

[85] Aldrees, R. M., Al-Gahtani, K. S., Alsugair, A. M., Aljadhai, S. I. & Alsanabani, N. Integrated value engineering and multi-criteria decision-making process in BIM framework for pipe materials selection. *KSCE J. Civ. Eng.* **29**, 100065. 10.1016/j.kscej.2024.100065 (2025).

[86] Saeed, M. et al. Sustainable selection of microwave absorbing materials: A green evaluation under interval-valued intuitionistic fuzzy environment. *Clean. Mater.* **11**, 100236. 10.1016/j.clema.2024.100236 (2024).

[87] Momena, A. F. Solution strategy for sustainable additive manufacturing design problem using Pythagorean fuzzy MCGDM methodology. *Complex Intell. Syst.* **10**, 3513–3539. 10.1007/s40747-023-01339-2 (2024).

[88] Sinha, A. K., Narang, H. K. & Bhattacharya, S. Experimental determination, modelling and prediction of sliding wear of hybrid polymer composites using RSM and fuzzy logic. *Arab. J. Sci. Eng.* **46**, 2071–2082. 10.1007/s13369-020-04997-3 (2021).

[89] Pamucar, D., Žižović, M. & Đuričić, D. Modification of the CRITIC method using fuzzy rough numbers. *Decis. Making Appl. Manag. Eng.* **5**, 362–371. 10.31181/dmame0316102022p (2022).

[90] Kang, D. et al. A novel MCDM approach to selecting a biodegradable dynamic plastic product: A probabilistic hesitant fuzzy set-based COPRAS method. *J. Environ. Manag.* **340**, 117967. 10.1016/j.jenvman.2023.117967 (2023). 10.1016/j.jenvman.2023.117967 37119624

[91] Nickel, V., Ottersbach, P., Reichert, R. & Schfer, M. In *International Conference on Sensor Networks, SENSORNETS.* 345–350. 10.5220/0004699103450350 (2014).

[92] Soni, A., Vellaisamy, M. & Veeman, D. Synergic effect of biofillers reinforcements and polylactic acid on the mechanical performance of biocomposites for structural applications. *Clean Technol. Environ. Policy*10.1007/s10098-025-03270-1 (2025).

[93] Soni, A., Yadav, P. C., Veeman, D. & Katiyar, J. K. Fruit waste-derived hybrid biofillers as a potential reinforcement in 3D printed biocomposites for building construction applications. *J. Manuf. Process.* **152**, 205–221. 10.1016/j.jmapro.2025.08.002 (2025).

[94] Jasmee, S., Omar, G., Othaman, S. S. C., Masripan, N. A. & Hamid, A. H. Interface thermal resistance and thermal conductivity of polymer composites at different types, shapes, and sizes of fillers: A review. *Polym. Compos.* **42**, 2629–2652. 10.1002/pc.26029 (2021).

[95] Sajith, S. Investigation on effect of chemical composition of bio-fillers on filler/matrix interaction and properties of particle reinforced composites using FTIR. *Compos. B Eng.* **166**, 21–30. 10.1016/j.compositesb.2018.11.141 (2019).

[96] Kuan, H. T. N., Tan, M. Y., Shen, Y. & Yahya, M. Y. Mechanical properties of particulate organic natural filler-reinforced polymer composite: A review. *Compos. Adv. Mater.* **30**, 26349833211007504. 10.1177/26349833211007502 (2021).

[97] Senthil Muthu Kumar, T. et al. Influence of fillers on the thermal and mechanical properties of biocomposites: an overview. *Biofibers Biopolym. Biocompos. Synth. Charact. Prop.*10.1007/978-3-030-40301-0_5 (2020).

[98] Inphonlek, S., Bureewong, N., Kotchapradit, S., Ruksakulpiwat, Y. & Ruksakulpiwat, C. Synergistic effects of hybrid bio-fillers and modified natural rubber on natural rubber composite properties. *Polymers* **17**, 632. 10.3390/polym17050632 (2025). 40076124 10.3390/polym17050632 PMC11902683

[99] Vallittu, P. K. High-aspect ratio fillers: Fiber-reinforced composites and their anisotropic properties. *Dent. Mater.* **31**, 1–7. 10.1016/j.dental.2014.07.009 (2015). 25088348 10.1016/j.dental.2014.07.009

[100] Gong, L.-X. et al. Polymer grafted reduced graphene oxide sheets for improving stress transfer in polymer composites. *Compos. Sci. Technol.* **134**, 144–152. 10.1016/j.compscitech.2016.08.014 (2016).

[101] Çallıoğlu, H., Sayer, M., Demir, E. & Ağır, İ. Effects of ceramic fillers on failure loads and failure mechanisms of laminated composites with two-parallel pin-loaded holes. *Mech. Adv. Mater. Struct.* **31**, 13412–13424. 10.1080/15376494.2023.2282107 (2024).

[102] Mehdikhani, M., Gorbatikh, L., Verpoest, I. & Lomov, S. V. Voids in fiber-reinforced polymer composites: A review on their formation, characteristics, and effects on mechanical performance. *J. Compos. Mater.* **53**, 1579–1669. 10.1177/0021998318772152 (2019).

[103] Vishal, K., Rajkumar, K., Sabarinathan, P. & Dhinakaran, V. Mechanical and wear characteristics investigation on 3D printed silicon filled poly (lactic acid) biopolymer composite fabricated by fused deposition modeling. *SILICON* **14**, 9379–9391. 10.1007/s12633-022-01712-9 (2022).

[104] Sakthi Balan, G. & Aravind Raj, S. Effect of additives on the performance of extruded polymer composites: A comprehensive review. *J. Thermoplast. Compos. Mater.* **38**, 810–853. 10.1177/08927057241261314 (2025).

[105] Bonferroni, C. Sulle medie multiple di potenze. *Bollettino dell’Unione Matematica Italiana* **5**, 267–270 (1950).

[106] Lendvai, L., Jakab, S. K. & Singh, T. Optimal design of agro-residue filled poly (lactic acid) biocomposites using an integrated CRITIC-CoCoSo multi-criteria decision-making approach. *Sci. Rep.* **15**, 11586. 10.1038/s41598-025-92724-z (2025). 40185808 10.1038/s41598-025-92724-z PMC11971358

[107] Diakoulaki, D., Mavrotas, G. & Papayannakis, L. Determining objective weights in multiple criteria problems: The critic method. *Comput. Oper. Res.* **22**, 763–770. 10.1016/0305-0548(94)00059-H (1995).

[108] Krishnan, A. R., Kasim, M. M., Hamid, R. & Ghazali, M. F. A modified CRITIC method to estimate the objective weights of decision criteria. *Symmetry* **13**, 973. 10.3390/sym13060973 (2021).

[109] Demir, S., Stappers, B., Kok, K. & Paterakis, N. G. Statistical arbitrage trading on the intraday market using the asynchronous advantage actor–critic method. *Appl. Energy* **314**, 118912. 10.1016/j.apenergy.2022.118912 (2022).

[110] Kildienė, S., Kaklauskas, A. & Zavadskas, E. K. COPRAS based comparative analysis of the European country management capabilities within the construction sector in the time of crisis. *J. Bus. Econ. Manag.* **12**, 417–434. 10.3846/16111699.2011.575190 (2011).

[111] Alinezhad, A., Khalili, J., Alinezhad, A. & Khalili, J. COPRAS method. *New methods and applications in multiple attribute decision making (Madm)*, 87–91 (2019).

[112] Zavadskas, E. K., Kaklauskas, A., Turskis, Z. & Tamošaitiene, J. Selection of the effective dwelling house walls by applying attributes values determined at intervals. *J. Civ. Eng. Manag.* **14**, 85–93. 10.3846/1392-3730.2008.14.3 (2008).

[113] Dhruva, S. et al. Selection of waste treatment methods for food sources: An integrated decision model using q-rung fuzzy data, LOPCOW, and COPRAS techniques. *Clean Technol. Environ. Policy*10.1007/s10098-025-03160-6 (2025).

[114] Kabeer, V. A., Maniyeri, R. & Anish, S. Multi-criteria decision-making techniques based optimum selection of phase change material and its implementation in a solar crop dryer for agricultural products. *J. Energy Storage* **137**, 118529. 10.1016/j.est.2025.118529 (2025).

[115] Adamu, M., Ibrahim, Y. E. & Raut, A. High-temperature performance evaluation of sustainable date palm fiber concrete with activated carbon: An MCDM and Weibull analysis approach. *Results Control Optim.*10.1016/j.rico.2025.100602 (2025).

